# Diagnosing type 2 diabetes using Hemoglobin A1c: a systematic review and meta-analysis of the diagnostic cutpoint based on microvascular complications

**DOI:** 10.1007/s00592-020-01606-5

**Published:** 2020-11-03

**Authors:** Alexandra E. Butler, Emma English, Eric S. Kilpatrick, Linda Östlundh, Hiam S. Chemaitelly, Laith J. Abu-Raddad, K. George M. M. Alberti, Stephen L. Atkin, W. Garry John

**Affiliations:** 1 Diabetes Research Center (DRC), Qatar Biomedical Research Institute (QBRI), Hamad Bin Khalifa University (HBKU), Qatar Foundation (QF), PO Box 34110, Doha, Qatar; 2 University East Anglia, Norwich, UK; 3 Sidra Medicine, Doha, Qatar; 4 College of Medicine and Health Sciences, United Arab Emirates University, Al Ain, UAE; 5 Infectious Disease Epidemiology Group, Weill Cornell Medicine-Qatar, Cornell University, Qatar Foundation-Education City, Doha, Qatar; 6 Imperial College, London, UK; 7 Royal College of Surgeons Ireland, Busaiteen, Bahrain; 8 Norfolk and Norwich University Hospital, Norwich, UK

**Keywords:** HbA1c, Type 2 diabetes, Microvascular complications

## Abstract

**Aims:**

Diabetic microvascular complications of retinopathy, nephropathy and neuropathy may occur at hemoglobin A1c levels (HbA1c) below the 6.5% (48 mmol/mol) diagnostic threshold. Our objective was to assess the validity of the HbA1c diagnostic cutpoint of 6.5% based upon published evidence of the prevalence of retinopathy, nephropathy and neuropathy as markers of diabetes.

**Methods:**

*Data Sources* PubMed, Embase, Cochrane, Scopus and CINAHL from 1990-March 2019, grey literature sources. *Study Selection* All studies reported after 1990 (to ensure standardized HbA1c values) where HbA1c levels were presented in relation to prevalence of retinopathy, nephropathy or neuropathy in subjects not known to have diabetes. *Data Extraction* Studies were screened independently, data abstracted, and risk of bias appraised. *Data Synthesis* Data were synthesized using HbA1c categories of < 6.0% (< 42 mmol/mol), 6.0–6.4% (42–47 mmol/mol) and ≥ 6.5% (≥ 48 mmol/mol). Random-effects meta-analyses were conducted for retinopathy, nephropathy and neuropathy prevalence stratified by HbA1c categories. Random-effects multivariable meta-regression was conducted to identify predictors of retinopathy prevalence and sources of between-study heterogeneity.

**Results:**

Pooled mean prevalence was: 4.0%(95% CI: 3.2–5.0%) for retinopathy, 10.5% (95% CI: 4.0–19.5%) for nephropathy, 2.5% (95% CI: 1.1–4.3%) for neuropathy. Mean prevalence when stratified for HbA1c < 6.0%, 6.0–6.4% and ≥ 6.5% was: retinopathy: 3.4% (95% CI: 1.8–5.4%), 2.3% (95% CI: 1.6–3.2%) and 7.8%(95% CI: 5.7–10.3%); nephropathy: 7.1% (95% CI: 1.7–15.9%), 9.6% (95% CI: 0.8–26.4%) and 17.1% (95% CI: 1.0–46.9%); neuropathy: 2.1% (95% CI: 0.0–6.8%), 3.4% (95% CI: 0.0–11.6%) and 2.8% (95% CI: 0.0–12.8%). Multivariable meta-regression showed HbA1c ≥ 6.5% (OR: 4.05; 95% CI: 1.92–8.57%), age > 55 (OR: 3.23; 95% CI 1.81–5.77), and African-American race (OR: 10.73; 95% CI: 4.34–26.55), to be associated with higher retinopathy prevalence. Marked heterogeneity in prevalence estimates was found across all meta-analyses (Cochran’s *Q*-statistic *p* < 0.0001).

**Conclusions:**

The prevalence of nephropathy and moderate retinopathy was increased in subjects with HbA1c values ≥ 6.5% confirming the high specificity of this value for diagnosing T2DM; however, at HbA1c < 6.5% retinopathy increased at age > 55 years and, most strikingly, in African-Americans, suggesting there may be excess microvascular complication prevalence (particularly nephropathy) in individuals below the diabetes diagnostic threshold.

**Electronic supplementary material:**

The online version of this article (10.1007/s00592-020-01606-5) contains supplementary material, which is available to authorized users.

## Introduction

The prevalence of diabetes has reached epidemic proportions globally, with 424.9 million affected adults (20–79 y), representing 8.8% of the global adult population. Current projections indicate that this figure will rise to 628.6 million by the year 2045, affecting almost 10% of the worldwide adult population [1]. Type 2 diabetes (T2DM) accounts for the vast majority (90–95%) of diabetes cases and is commonly characterized by the inability of pancreatic beta cells to meet the demand for insulin secretion due to a relative deficit of functional beta cells in a setting of peripheral insulin resistance. There has been much debate over the years as to how T2DM should be diagnosed; what should be measured, and the diagnostic targets have changed, being refined as our understanding of the disease has improved along with improvement in analytical methods. The diagnostic criteria for T2DM are established [2], but it is clear that a continuum in blood glucose level exists from normoglycemia to frank diabetes. As such, in 1997 and 2003, the Expert Committee on Diagnosis and Classification of diabetes mellitus recognized a cohort of subjects whose glucose levels did not meet the criteria for diabetes but were too high to be considered as normal [3, 4]; this “prediabetic” group exhibited impaired fasting glucose (IFG) [fasting plasma glucose (FPG) levels of 100–125 mg/dL (5.6–6.9 mmol/L) and/or impaired glucose tolerance (IGT)] defined as a 2-h plasma glucose following a 75 g oral glucose tolerance test (OGTT) of 140–199 mg/dL (7.8–11.0 mmol/L)] and represents individuals at high risk for development of T2DM.

Hemoglobin A1c (HbA1c) is considered key for assessing glycemic control in patients known to have diabetes, and several prospective studies using HbA1c have shown a strong, continuous association between HbA1c and the development of diabetes and complications [5–7]. An International Expert Committee (IEC) recommended an HbA1c level of 6.5% (48 mmol/mol) as the diagnostic threshold for T2DM diagnosis [8], purporting that individuals with HbA1c levels above this cutoff have a much higher probability of having retinopathy than those below. Both the American Diabetes Association (ADA) and, subsequently, the World Health Organization (WHO) endorsed this opinion, which was entirely based upon the risk of observing diabetic retinopathy, without consideration for other diabetic microvascular complications; however, the subject has to have had diabetes for a period of time for microvascular complications to develop that may occur at a lower HbA1c than 6.5%.

Key studies contributing to the IEC/ADA/WHO diagnostic threshold decision were the results of the cross-sectional Evaluation of Screening and Early Detection Strategies for Type 2 Diabetes and Impaired Glucose Tolerance (DETECT-2) study [9] and three epidemiological studies undertaken in the 1990s on Pima Indians, Egyptians and US subjects enrolled in the National Health and Nutrition Examination Survey (NHANES) study [3, 10, 11].

The current HbA1c threshold was based on data available at the time of these groups’ reports and, even at the time, there was debate as to whether a level of 6.5% (48 mmol/mol) may be too high since, in many studies, it identified fewer patients as having diabetes than the traditional blood glucose criteria [12]. Since then, a number of studies have been performed with the aim of better characterizing the HbA1c threshold for prevalent or incident retinopathy. The studies prior to 2013 were summarized in a publication by Kowall and Rathmann who looked at retinopathy, nephropathy and neuropathy [13]; since this time, there have been a number of relevant publications which can now be considered.

The key aims of this study were to perform a contemporary systematic review and meta-analysis to verify the HbA1c cutpoint of 6.5% (48 mmol/mol) for the diagnosis of T2DM using currently available retinopathy data and, secondly, to extend the analysis to establish the prevalence of nephropathy and neuropathy at differing levels of HbA1c.

## Methods

### Data sources and search strategy

This systematic review was guided by the Cochrane Collaboration Handbook [14], and followed the Preferred Reporting Items for Systematic Reviews and Meta-analyses (PRISMA) guidelines [15]. The PRISMA checklist is shown in Supplementary Table 1. A comprehensive systematic search for literature was conducted in the academic databases PubMed, Embase, Cochrane, Scopus, CINAHL and in sources for grey literature in October 2019 (Supplementary Table 2).

**Table 1:** Studies included for assessment of HbA1c level and incidence of retinopathy

	Authors	Title	Age [years]	Mid-point age [years]	HbA1c [%]	Mid-point HbA1c [%] cutoff if only range given	Retinopathy diagnosis/grading
1	Colagiuri et al. [9]; studies pooled from 5 countries (USA × 4, Australia × 2, India, Japan, Singapore)	Glycemic thresholds for Diabetic retinopathy	20–79 years	50	6		9 studies from 5 countries. Retinopathy classified as present or absent for initial analysis; where data available, further classified into minimal non-proliferative diabetic retinopathy (NPDR), mild NPDR, moderate NPDR, severe NPDR or proliferative diabetic retinopathy (PDR) based on information provided by individual studies using the modified Airlie House classification levels, modified Early Treatment Diabetic Retinopathy Study levels or the Fukuda standard. Levels 14–20 indicate minimal NPDR; levels 30–35 or Fukuda standard A1 indicate mild NPDR; levels 40–47 or the Fukuda standard A2 indicate moderate NPDR; levels 50–53 or Fukuda standard A3 indicate severe NPDR; levels 60–90 or Fukuda standards A4 and B1-B4 indicate PDR. Final retinopathy grading was based on diagnosis in more severely affected eye. The primary outcome used was diabetes-specific retinopathy, defined as moderate or severe
					6.4		
					6.5		
2	Engelgau et al. [11], Egypt	Comparison of fasting and 2-h glucose and HbA1c levels for diagnosing diabetes	> 20 years; mean 45 years Egyptians	45	6.4		Bilateral retinal photographs of the fundus through dilated pupils. Photographs graded with a modified Airlie House classification scheme for diabetic retinopathy; retinopathy present when there were retinal microaneurysms either alone or with non-proliferative changes (hard or soft exudates, intra-retinal microangiopathy or retinal hemorrhages), pre-proliferative or proliferative changes or vitreous hemorrhage
					6.5		
3	Ito et al. [27], Japan	Importance of OGTT for diagnosing diabetes mellitus based on prevalence and incidence of retinopathy	Mean age 59.2 (SD 9.5) years	59.2	5.5		Retinopathy was examined by bilateral fundus photography. No further details given
					5.8		
					6		
					6.2		
					6.5		
4	Tapp et al. [28] Australia	Diagnostic thresholds for diabetes: the association of retinopathy and albuminuria with glycemia; AusDiab Study	> 25 years; mean age 59 (men 60, women 58) Australians		5.7		Bilateral retinal photographs. Retinopathy defined according to a simplified version of the Wisconsin grading system (based on grading of worst eye). Diabetic retinopathy defined as presence of at least one definite retinal hemorrhage and/or microaneurysm
					6.1		
					6.5		
5	Aidenloo [29], Iran	Optimal glycemic and hemoglobin A1c thresholds for diagnosing diabetes based on prevalence of retinopathy in an Iranian population	40–81 years; mean 54.7 ± 8.4 years Iranians		5.5		Bilateral retinal photographs through dilated pupils. Assessed according to the international clinical DR severity scale: 5 levels: no retinopathy, mild non-proliferative retinopathy (NPDR), moderate NPDR, severe NPDR; diagnosis according to worst eye. Presence of DR defined as presence of moderate or severe non-proliferative DR or proliferative DR in either eye
					6		
					6.1		
					6.2		
					6.3		
					6.4		
					6.5		
6	Almdal [30], Saudi Arabia, Algeria and Portugal	Glycemic threshold for diabetes specific retinopathy among individuals from Saudi Arabia, Algeria and Portugal	30–75 years (mean age varied from 44.6 years in Saudi Arabia to 60.8 years in Portugal)	52.5	6–6.4	6.2	2 digital photographs of the retina in each eye. Images assessed according to a modified ETDRS scale standard; presence of any retinopathy-related abnormalities in at least one eye or presence of diabetes-specific retinopathy in at least one eye (defined as presence of moderate or severe non-proliferative retinopathy)
					6.5–6.9	6.5	
7	Bertelsen [31], Norway	Sex differences in risk factors for retinopathy in non-diabetic men and women: the Tromso Eye study	38–87 years; median 62 years Norwegian	62	5.66 ± 0.36 (males)	5.66	Bilateral retinal photographs of the fundus through dilated pupils. All images graded for retinopathy according to 'The International Clinical Diabetic Retinopathy and Diabetic Macular Edema Disease Severity Scales.' Microaneurysms, hemorrhages and cotton wool spots were quantified
					5.62 ± 0.36 (females)	5.62	
8	Bower [32], USA	No ethnic difference in the Association of glycated hemoglobin with retinopathy	> 40 years	56.7 (White)	5.7–6.4		Two 45 degree non-mydriatic digital images were obtained from each eye. Retinopathy determined as described by the Early Treatment Diabetic Retinopathy Study (ETDRS); any retinopathy defined as an ETDRS level of 14 or higher in the worse eye
			Mean age: Non-Hispanic White 56.7 ± 0.5; Non-Hispanic Black 53.5 ± 0.4; Hispanic American 51.9 ± 0.4)	53.5 (Black)		6.1	
						6.1	
				55.1 (White + Black)		6.1	
						6.5	
						6.5	
						6.5	
9	Cheng [33], USA	Association of A1C and fasting plasma glucose levels with diabetic retinopathy prevalence in the US population	> 40 years	56	5.5–6.4	6	Two 45 degree non-mydriatic digital images were obtained from each eye. Retinopathy lesions graded according to a modified Airlie House classification system, as used in the ETDRS; retinopathy > 14, no retinopathy < 14 on ETDRS severity level
			Mean age 56 years			6.5	
10	Cho [34], South Korea	Optimal HbA1c cutoff for detecting diabetic retinopathy	40–69 years Mean age 63.3 ± 8.6 South Korea	63.3	5.7		Single-field 45 degrees non-mydriatic fundus photography of each eye. Retinopathy classified as mild non-proliferative diabetic retinopathy (NPDR), moderate NPDR, severe NPDR, proliferative diabetic retinopathy (PDR) or prior panretinal photocoagulation ((PRP) according to the International Clinical Diabetic retinopathy Disease Severity Scale
					5.8		
					5.9		
					6		
					6.1		
					6.3		
					6.5		
11	Fukushima [35], Japan	Prevalence of retinopathy and its risk factors in a Japanese population	Mean age: retinopathy absent 51 ± 8; retinopathy present 53 ± 7 years	52		6.1	Two retinal photographs per subject used for retinal grading. Retinopathy defined by the presence of microaneurysms, any retinal hemorrhage, soft and hard exudates
			Japanese			6.5	
12	Selvin [6], USA	Glycated hemoglobin and the risk of kidney disease and retinopathy in adults with and without diabetes	Mean age 56.7 ± 2.7 USA: White and Black, results not separated	56.7	5.7–6.4	6.1	One 45 degree non-mydriatic digital images obtained from a random eye. Retinopathy lesions graded according to a modified Airlie House classification system, as used in the ETDRS; retinopathy > 14, no retinopathy < 14 on ETDRS severity level. Mild retinopathy usually consists of one or two microaneurysms or small hemorrhages; moderate or severe retinopathy consists of both microaneurysms and hemorrhages, often accompanied by hard or soft exudates, intraretinal microvascular abnormalities, venous beading or less commonly vascular proliferative changes
						6.5	
13	Park [36] , Korea	Glycemic and hemoglobin A1c thresholds for detecting diabetic retinopathy: the fifth Korea national Health and Nutrition Examination (2011)	> 19 years Mean age 44.3 ± 0.4 years Korean	44.3	6.4		Single-field 45 degrees non-mydriatic fundus photography of each eye. Retinopathy severity score assigned according to the ETDRS severity scale. DR defined as presence of one or more retinal microaneurysms or blot hemorrhages with or without more severe lesions (hard exudates, soft exudates, intraretinal microvascular abnormalities, venous bleeding, new retinal vessels and fibroproliferations) The final retinopathy grading for each participant was based on the diagnosis in the more severely affected eye
					6		
					6.1		
					6.2		
					6.3		
					6.4		
					6.5		
14	Lamparter [37], Germany	Prevalence and associations of diabetic retinopathy in a large cohort of prediabetic subjects: the Gutenberg Health study	35–74 years, Germany Mean age 60.0 ± 9.1 years	60	5.7–6.4	6	3 images of each eye. Retinopathy lesions graded according to definitions used in the Early Treatment Diabetic Retinopathy Study (ETDRS) classification system. DR defined as one or more microaneurysms or blot hemorrhages with or without hard exudates, cotton wool spots, intraretinal microvascular abnormalities, venous beading, retinal neovascularizations and pre-retinal/vitreous hemorrhage
15	Metcalf [38], New Zealand	HbA1c in relation to incident diabetes and diabetes-related complications in non-diabetic adults at baseline	Mean age 57.6 years New Zealand	57.6	5.8–6	5.9	Not specified
					6.1–6.2	6.2	
						6.6	
16	Okosun [40], USA	Diagnostic performance of glycated hemoglobin for diabetic retinopathy in non-diabetic older overweight/obese African-Americans	Mean age 62.6 ± 8.2 years African-American	62.6	5.5–5.9	5.7	As part of NHANES questionnaire, subjects asked if they were ever told they had retinopathy? Digital retinal photography assessed by modified Airlie House classification system as used in the Early Treatment Diabetic Retinopathy Study. In this study, diabetic retinopathy defined if participant answered the survey questionnaire positively, if there were 1 or more microaneurysms, or if more severe forms of retinopathy were present
					6.0–6.4	6.2	
					6.5–6.9	6.7	
17	Pang [41] , China	Determination of diabetic retinopathy prevalence and associated risk factors in Chinese diabetic and pre-diabetic subjects: Shanghai diabetic complications study	Mean age 60.7 ± 11.0 years (20.9–93.3 years)	60.7	5.8 ± 0.6%	5.8	Retinal photography done both eyes. DR was graded according to Diabetic Retinopathy Disease Severity Scale: Grade 0 = no abnormality; Grade 1 = mild non-proliferative (microaneurysms only); Grade 2 = moderate non-proliferative (more than just microaneurysms but less than Grade 3; Grade 3 = severe non-proliferative retinopathy; Grade 4 = proliferative retinopathy
18	Sabanayagam [42] , Chinese, Malay, Indian	Diagnosis of Diabetes Mellitus Using HbA1c in Asians: Relationship between HbA1c and retinopathy in a multiethnic population	Mean age: Chinese 54.6 ± 11.7 years, Malays 57.0 ± 11.5 years, Indians 56.4 ± 10.3 years	56 (overall mean); 54.6 (Chinese), 57.0 (Malay), 56.4 (Indian)	HbA1c 6.2%: Chinese	6.2	Digital retinal photographs, 2 for each eye. Retinopathy present if any characteristic lesion as defined by ETDRS severity scale was present: microaneurysms, hemorrhages, cotton wool spots, intraretinal microvascular abnormalities, hard exudates, venous beading and new vessels. For each eye, a retinopathy severity score was assigned using the modified Airlie House Classification System; based on the severity score of the worst eye, "moderate" retinopathy corresponded to a level above 43 as the outcome for the current analysis (this level felt to be more diabetes specific, as mild levels of retinopathy are found in 10–15% of the general population and are less specific for diabetes)
					HbA1c 6.3%: Chinese	6.3	
					HbA1c 6.4%: Chinese	6.4	
					HbA1c 6.7%: Chinese	6.7	
					HbA1c 6.2%: Malay	6.2	
					HbA1c 6.3%: Malay	6.3	
					HbA1c 6.4%: Malay	6.4	
					HbA1c 6.7%: Malay	6.7	
					HbA1c 6.2%: Indians	6.2	
					HbA1c 6.3%: Indians	6.3	
					HbA1c 6.4%: Indians	6.4	
					HbA1c 6.7%: Indians	6.7	
19	Sabanayagam [43], Malay	Relationship between glycated hemoglobin and microvascular complications: is there a natural cutpoint for the diagnosis of diabetes?	40–80 years Malay adults in Singapore	60	5.6–6.0	5.6	2 Digital photographs per eye following ETDRS protocol; Retinopathy defined as (1) any retinopathy: severity score of level 15 and above in worse eye according to ETDRS adaptation of modified Airlie House Classification System (corresponds to the presence of any of the following: microaneurysms, hemorrhages, cotton wool spots, intraretinal microvascular abnormalities, hard exudates, venous beading and new vessels). (2) mild retinopathy defined as ETDRS score > 20 (3) moderate retinopathy severity score > 43
						5.8	
						6.1	
						6.5	
20	Tsugawa [44], Japan	New Diabetes Diagnostic threshold of hemoglobin A1c and the 3-year incidence of retinopathy	> 21 years Mean age 51.0 ± 11.7 years Japanese	51	5.5–5.9	5.7	Digital photographs both eyes. Retinopathy defined as presence of hard exudates, cotton wool spots, retinal hemorrhage, or more severe forms of retinopathy
					6.0–6.4	6.2	
					6.5–6.9	6.7	
21	Massin [45], France	Hemoglobin A1c and fasting plasma glucose levels as predictors of retinopathy at 10 years: the French DESIR study	30–65 years Mean age 52 years French		5.5–5.9	5.7	3 retinal photographs per eye. Graded using simplified version of Wisconsin protocol. DR defined as microaneurysms, hemorrhages, exudates, cotton wool spots, intramicrovascular abnormalities, venous bleeding or new vessels (worse eye used)
					6.0–6.4	6.2	
					6.5–6.9	6.7	
22	Tsugawa [46], USA	Should the Hemoglobin A1c Diagnostic cutoff differ between Blacks and Whites?	> 40 years Mean age: White 57.1 ± 0.4 years, Black 54.4 ± 0.4 years		5.5–5.9 White		2 digital photographs per eye. Graded using the modified Airlie House classification system as in the ETDRS. DR defined as 1 or more microaneurysm or more severe forms of retinopathy
					6.0–6.4 White		
					6.5–6.9 White		
					5.5–5.9 Black		
					6.0–6.4 Black		
					6.5–6.9% Black

**Table 2:** Studies included for assessment of HbA1c level and incidence of nephropathy

	Authors	Title	Age	Mid-point Age	HbA1c	Mid-point HbA1c cutoff if only range given
1	Tapp et al. [49] Australia	Diagnostic thresholds for diabetes: the association of retinopathy and albuminuria with glycemia; AusDiab Study	> 25 years	59	5.6	5.6
			Mean age 59 years Australia		5.8	5.8
					6.1	6.1
					6.5	6.5
2	Metcalf [38], New Zealand	HbA1c in relation to incident diabetes and diabetes-related complications in non-diabetic adults at baseline	Mean age 57.6 years New Zealand	57.6	5.8–6.0	5.9
					6.1–6.2	6.2
					6.3–6.7	6.5
3	Toulis et al. [47] China Glycated hemoglobin, albuminuria and surrogate markers of macrovascular disease; the Guangzhou Biobank Cohort Study	Glycated hemoglobin, albuminuria and surrogate markers of macrovascular disease; the Guangzhou Biobank Cohort Study	Mean age: HbA1c < 5.7, 57 + -9 years; 5.7–6.4, 58 + -9 years; > 6.5, 60 + -10 years Chinese	58	< 5.7%	5.6
					5.7–6.4%	6
					> 6.5	
4	Xing et al. [48], USA	Association of pre-diabetes by fasting glucose and/or HbA1c levels with subclinical atherosclerosis an impaired renal function; observations from the Dallas Heart Study	Mean age 49 years USA African-American (50%), Caucasian, Hispanic	49	5.6	5.6
					5.7–6.4	6

Pre-searches to identify relevant search terms, search strategies and information sources were performed in May–October 2019. PubMed’s MeSH was used to systematically identify search terms that encompass the variations in terminology for glycated hemoglobin, including thresholds or cutpoints, as well as terms that describe diabetic microvascular complications (Tables 1, 2, 3**)**. The search strategy developed in PubMed was replicated in all databases with eventual search terms or technical variations documented (Supplementary Table 2). The PRESS peer review of electronic search strategies: 2015 guideline statement was used to peer review the search string [16].

**Table 3:** Studies included for assessment of HbA1c level and incidence of neuropathy

	Authors	Title	Age	Mid-point Age	HbA1c	Mid-point HbA1c cutoff if only range given
1	Metcalf PA [38], New Zealand	HbA1c in relation to incident diabetes and diabetes-related complications in non-diabetic adults at baseline	Mean age 57.6 years New Zealand	57.6	5.8–6.0	5.9
					6.1–6.2	6.1
					6.3–6.7	6.5
2	Kurisu et al. [50] poster, Japan	Polyneuropathy or neuropathic pain did not increase at prediabetic stage in a Japanese population	Mean age 62.1 years Japanese	62.1	5.9	
					6.2	
3	Tapp [49] Australia	Foot complications in type 2 diabetes: an Australian population-based study	> 25 years Mean age: neuropathy 73 ±10 years; no neuropathy 62 ±12 years Australia	66.5	ND IFG/IGT	5.9
						6.2
					Diabetic	6.5

All selected search terms were searched in a combination of “Abstract” and “Article Title” (alternatively “Topic” or “Title, Abstract and Keyword”) and in MeSH/Subject Headings/Thesaurus when available. A publication year filter to include studies from 1990 up to the search date was applied to account for the predominant lack of standardized/harmonized HbA1c measurement before this time. In order to ensure literature saturation and inclusion of pre-indexed materials, no additional filters or limitations were included.

In addition to the search in academic databases, Open Grey, Clinical Trials.gov, The New York Academy of Medicine-Grey Literature Report and ProQuest Dissertation and Theses were searched for grey literature. Hand screening of the references lists of all studies selected to be included in the review was also conducted.

A full search log including search technical details, results and notes about search term variations/translations for all databases can be found in Supplementary Table 2.

A review protocol for this study was registered in the Prospero international prospective register of systematic reviews: https://www.crd.york.ac.uk/prospero/display_record.php?RecordID=99410PROSPERO2018CRD42018099410.

### Study selection

Search results were imported into the Covidence systematic review tool where duplicate publications were identified and duplicates excluded. Study eligibility was independently determined by two investigators (AEB and SLA). Where there was a discrepancy, both investigators revisited the publication in question, discussed the results with the group and re-entered their decision into the database.

The criterion for inclusion was for a publication to report the prevalence of microvascular complications by levels of HbA1c. More specifically, studies were only considered if they provided a prevalence measure for a microvascular complication stratified by HbA1c of < 6% (< 42 mmol/mol), 6–6.4% (42–47 mmol/mol) and ≥ 6.5% (48 mmol/mol) in the same study. The reason for that is to ensure rigor in the association with microvascular complications. Exclusion criteria were studies that did not report microvascular complications by different HbA1c level and those published before 1990.

For terminology, a “publication” is a document containing a relevant outcome measure, while a “study” indicates all details pertaining to a specific outcome measure—one publication may contribute multiple studies. A study, such as “prevalence of retinopathy,” could include multiple stratified “measures,” such as prevalence by age group.

### Data extraction and quality assessment

Following screening of records for eligibility, eligible studies had full-text screening (Supplementary Figure 1). Extracted variables included: author(s), publication title, year(s) of data collection, publication year, country of origin, country of survey, study design, study sampling procedure, study population and its characteristics (e.g., sex, age and ethnicity), sample size, HbA1c measurement method, HbA1c stratification outcome measures, complications, retinal photography method, number of eyes photographed, determination of albumin creatinine ratio, measure of peripheral neuropathy. Two investigators (AEB and SLA) independently assessed the full-text articles and determined the eligibility of studies for inclusion in the systematic review.

### Quality assessment

Risk of bias (ROB) and precision assessments were performed for all studies included in the review. Guided by the Cochrane approach [17], studies were classified as having “low” vs. “high” ROB on two quality domains assessing (1) consistency in HbA1c diagnostic measurement across all study participants (consistent vs. not consistent) and (2) rigor of sampling methodology (probability-based vs. non-probability-based). Studies with unavailable information for any given domain were classified as having “unclear” ROB for that domain. Studies including at least 100 participants were considered as having higher precision. For a prevalence of microvascular complications of 1% (see prevalence by HbA1c in Table 1) and a sample size of 100, the 95% confidence interval (95% CI) is 0–5%, an acceptable CI for the prevalence of microvascular complications (a lower sample size is needed to detect a higher prevalence). Results of the quality assessment are shown in Supplementary Tables 3 and 4.

**Table 4:** Results of meta-analyses of studies reporting complications of diabetes in patients with different HbA1c (%)

	Studies	Sample		Prevalence (%)				Heterogeneity measures		
	Total *N*	Tested	Number positive	Median	Range	Pooled mean	95% CI	*Q*^a^ (*p* value)	*I*^2^^b^ (%, 95% CI)	Prediction interval^c^ (95%)
Retinopathy										
HbA1c ≤ 5.6	5	25,184	1047	5.00	0.90–15.90	5.19	0.50–14.33	1666.8 (*p* < 0.0001)	99.8 (99.7–99.8)	0.00–57.22
HbA1c = 5.7	7	11,896	356	5.10	0.00–14.40	4.22	1.95–7.25	226.1 (*p* < 0.0001)	97.3 (96.1–98.2)	0.00–18.26
HbA1c = 5.8–5.9	6	26,852	305	1.03	0.32–6.08	1.53	0.61–2.82	163.4 (*p* < 0.0001)	96.9 (95.2–98.1)	0.00–7.82
HbA1c = 6.0	6	22,723	352	0.86	0.10–8.10	1.91	0.39–4.49	322.4 (*p* < 0.0001)	98.4 (97.8–98.9)	0.00–16.16
HbA1c = 6.1	9	13,345	719	5.32	0.20–11.60	4.85	2.35–8.17	487.1 (*p* < 0.0001)	98.4 (97.8–98.8)	0.00–21.16
HbA1c = 6.2	13	49,100	599	1.20	0.02–14.80	1.95	1.24–2.82	387.8 (*p* < 0.0001)	96.9 (95.8–97.7)	0.04–6.24
HbA1c = 6.3–6.4	4	6754	19	0.30	0.10–0.62	0.24	0.13–0.39	3.02 (*p* = 0.3879)	0.8 (0.0–84.8)	0.03–0.60
HbA1c = 6.5	16	32,348	1793	6.44	0.40–22.20	8.28	4.46–13.10	2074.57 (*p* < 0.0001)	99.3 (99.2–99.4)	0.00–34.97
HbA1c ≥ 6.6	9	23,553	1133	5.14	2.99–48.30	5.49	4.18–6.95	110.2 (*p* < 0.0001)	92.7 (88.4–95.5)	1.84–10.76
HbA1c < 6.0	18	63,932	1708	2.65	0.00–15.90	3.41	1.84–5.42	2304.8 (*p* < 0.0001)	99.3 (99.1–99.4)	0.00–16.13
HbA1c = 6–6.4	32	91,922	1689	1.15	0.02–14.80	2.40	1.65–3.29	1869.8 (*p* < 0.0001)	98.3 (98.1–98.6)	0.00–9.35
HbA1c ≥ 6.5	24	55,901	2926	5.58	0.40–48.30	7.97	5.70–10.57	2194.7 (*p* < 0.0001)	99.0 (98.8–99.1)	0.21–24.18
Overall	74	211,755	6323	4.04	0.00–48.30	4.08	3.22–5.04	7647.0 (*p* < 0.0001)	99.0 (99.0–99.1)	0.00–15.10
Nephropathy										
HbA1c < 6.0*	5	11,959	637	11.20	1.02–19.70	7.87	2.55–15.73	648.9 (*p* < 0.0001)	99.4 (99.2–99.5)	0.00–48.78
HbA1c = 6.0–6.4	4	8677	672	12.42	0.92–18.50	9.56	0.75–26.41	1013.6 (*p* < 0.0001)	99.7 (99.6–99.8)	0.00–97.71
HbA1c ≥ 6.5*	3	15,184	2413	26.0	1.85–32.60	17.10	0.96–46.85	3076.4 (*p* < 0.0001)	99.9 (99.9–99.9)	0.00–100.00
Overall	12	35,820	3722	11.20	0.92–32.60	10.51	4.40–18.82	5442.1 (*p* < 0.0001)	99.8 (99.8–99.8)	0.00–52.99
Neuropathy										
HbA1c < 6.0	3	6545	41	2.80	0.22–4.98	2.06	0.02–6.76	59.6 (*p* < 0.0001)	96.6 (93.1–98.4)	0.00–100.00
HbA1c = 6.0–6.4	3	6436	82	5.56	0.23–8.45	3.39	0.00–11.55	150.0 (*p* < 0.0001)	98.7 (97.7–99.2)	0.00–100.00
HbA1c ≥ 6.5	2	8157	69	3.80	0.50–7.09	2.81	0.00–12.77	64.2 (*p* < 0.0001)	98.4 (96.6–99.3)	–
Overall	8	21,138	192	3.89	0.22–8.45	2.47	1.13–4.26	280.1 (*p* < 0.0001)	97.5 (96.4–98.3)	0.00–10.77

A minimum of two studies was necessary to conduct a meta-analysis

*Imputation of sample size at the median of studies in that category was performed for two studies to avoid loss of information

^a^*Q*: the Cochran’s *Q* statistic is a measure assessing the existence of heterogeneity in effect size (here, prevalence) across studies

^b^*I*^2^: a measure assessing the magnitude of between-study variation that is due to differences in effect size (here, prevalence) across studies rather than chance

^c^Prediction interval: a measure estimating the 95% interval of the distribution of true effect sizes (here, prevalence measures)

## Data synthesis and analysis

### Statistical analysis

#### Meta-analysis methodology

Meta-analyses were conducted to estimate the pooled mean of diabetes complications in relation to the stratified HbA1c values (extracted overall outcome measures for a specific HbA1c category, such as 6.0–6.4, were substituted with stratified measures such as by sub-levels of HbA1c, that is 6.0, 6.1, 6.2…, or population characteristics). Forest plots were constructed to visualize prevalence measures and 95% confidence intervals (CIs) associated with each of the three outcomes of interest (retinopathy, nephropathy and neuropathy). Estimates for the pooled mean prevalence and 95% CIs were then calculated using random-effects meta-analyses. Here, variances of prevalence measures were first stabilized using a Freeman–Tukey-type arcsine square-root transformation [18, 19]. Inverse variance weighting [19, 20] was subsequently applied prior to pooling estimates using a DerSimonian–Laird random-effects model [21]. This model assumes a normal distribution for true effect sizes (prevalence) across studies and thus factors in sampling variation and true heterogeneity in effect size [22].

A heterogeneity assessment was further conducted using Cochran’s *Q* statistic to confirm existence of heterogeneity across studies and I^2^ to quantify magnitude of between-study variation that is due to true differences in effect size rather than chance [22, 23].

Meta-analyses were implemented in R version 3.4.2 [24].

#### Meta-regression methodology

Associations with prevalence and sources of between-study heterogeneity were identified using random-effects univariable and multivariable meta-regression analyses. Predictors considered a priori included: HbA1c levels, age and race. Factors associated with prevalence at *p* value ≤ 0.10 in univariable analysis were eligible for inclusion in the multivariable model. In the latter, a *p* value ≤ 0.10 but > 0.05 was considered as showing “good evidence” for an association with prevalence, while a *p* value ≤ 0.05 indicated strong evidence for an association with prevalence.

The magnitude of the association between these predictors and prevalence was determined by calculating, in the univariable analysis, odds ratios (ORs) and their associated 95% CIs, and in the multivariable analysis, by calculating adjusted odds ratios (AORs) and their associated 95% CIs.

Meta-regressions were conducted in Stata/SE version 13 [25] using the metareg package [26].

## Results

### Literature search

A total of 12,845 references were identified through the literature search and uploaded to the systematic review software Covidence for de-duplication and screening. 9370 references (9324 from the database search and 46 from the grey literature search) qualified for title and abstract screening after de-duplication. The screening process in Covidence software was blinded. Of the 9370 references, 9206 records were excluded. 164 full-text articles were then assessed for eligibility; of those 135 were excluded because of insufficient details of the relationship of the microvascular complication to the HbA1c values less than 6.5% (48 mmol/mol). Twenty nine publications remained eligible for inclusion in the systematic review, 22 relating to retinopathy (Table 1) [6, 9, 11, 27–46]; 4 relating to nephropathy (Table 2) [28, 38, 47, 48] and 3 relating to neuropathy (Table 3) [38, 49, 50]. No additional relevant references were identified in the hand searching of the reference lists of the 29 included studies. The retinopathy studies contributed 74 stratified measures for the quantitative meta-analysis according to HbA1c as detailed above.

### Study characteristics

The definitions of retinopathy, nephropathy and neuropathy used in each study are shown in Tables 1, 2 and 3, respectively.

Diabetic retinopathy, defined as diabetes-related damage to the retina, is classified into early stage non-proliferative diabetic retinopathy (NPDR, comprising microaneurysms and macular edema) and second-stage proliferative diabetic retinopathy (PDR, comprising neovascularization and vitreous hemorrhages) and fundal photography is required for diagnosis [51]. HbA1c was stratified into the following categories in each study: < 6% (< 42 mmol/mol),  = 6–6.4% (42–47 mmol/mol) and  ≥ 6.5% (≥ 48 mmol/mol).

**Fig. 1:**
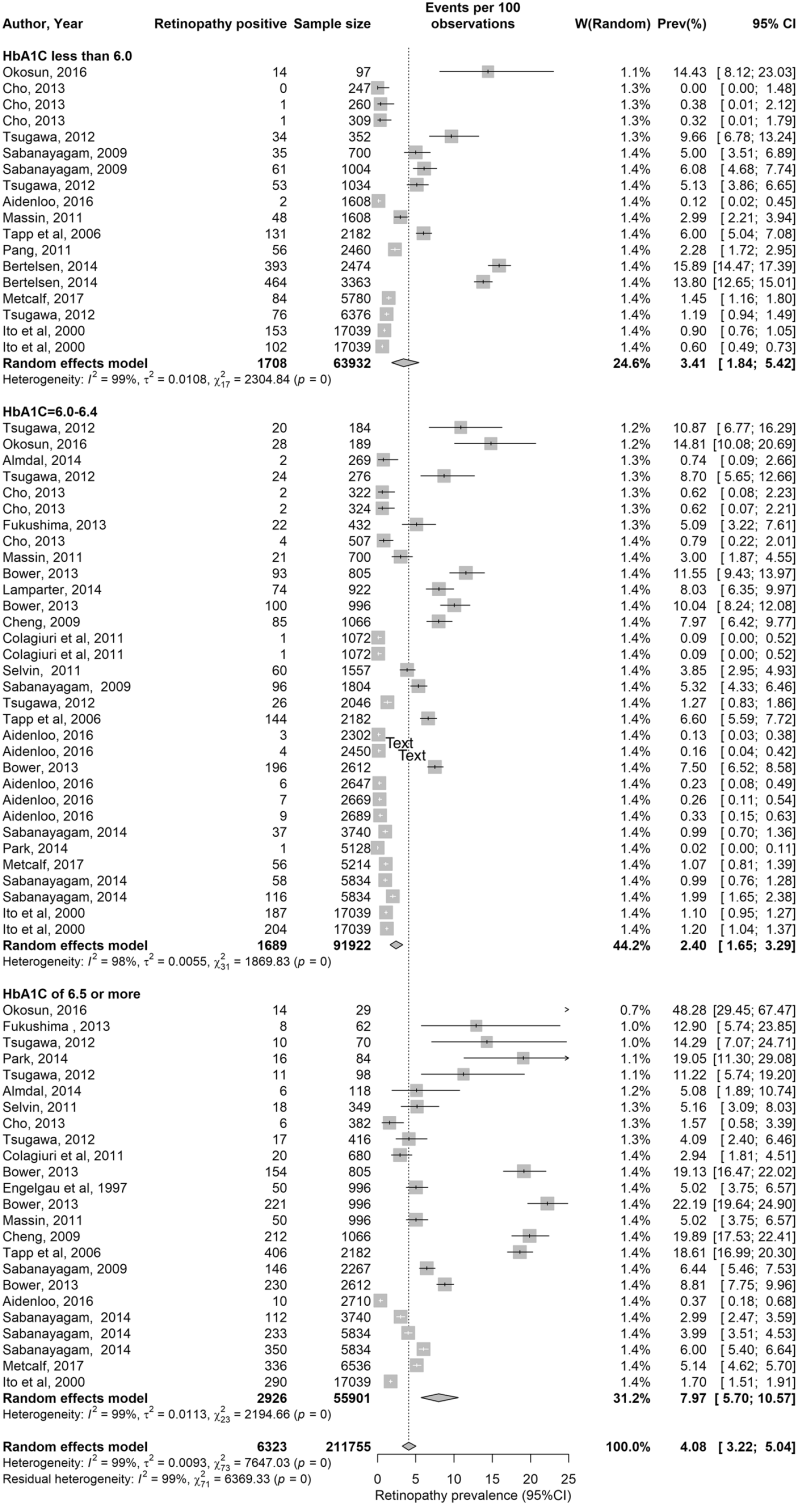
Forest plot showing results of the meta-analysis for retinopathy prevalence. Retinopathy prevalence stratified by HbA1c levels

The forest plot for retinopathy is shown in Fig. 1. The median prevalence of retinopathy was overall 4.0% (range: 0–48.3%), but varied according to HbA1c levels (Table 4). It was 2.7% (range: 0–15.9%) for HbA1c < 6.0% (< 42 mmol/mol), 1.2% (range: 0.2–14.8%) for HbA1c 6.0–6.4% 42–47 mmol/mol) and 5.6% (range: 0.4–48.3%) for HbA1c ≥ 6.5% (≥ 48 mmol/mol).

**Fig. 2:**
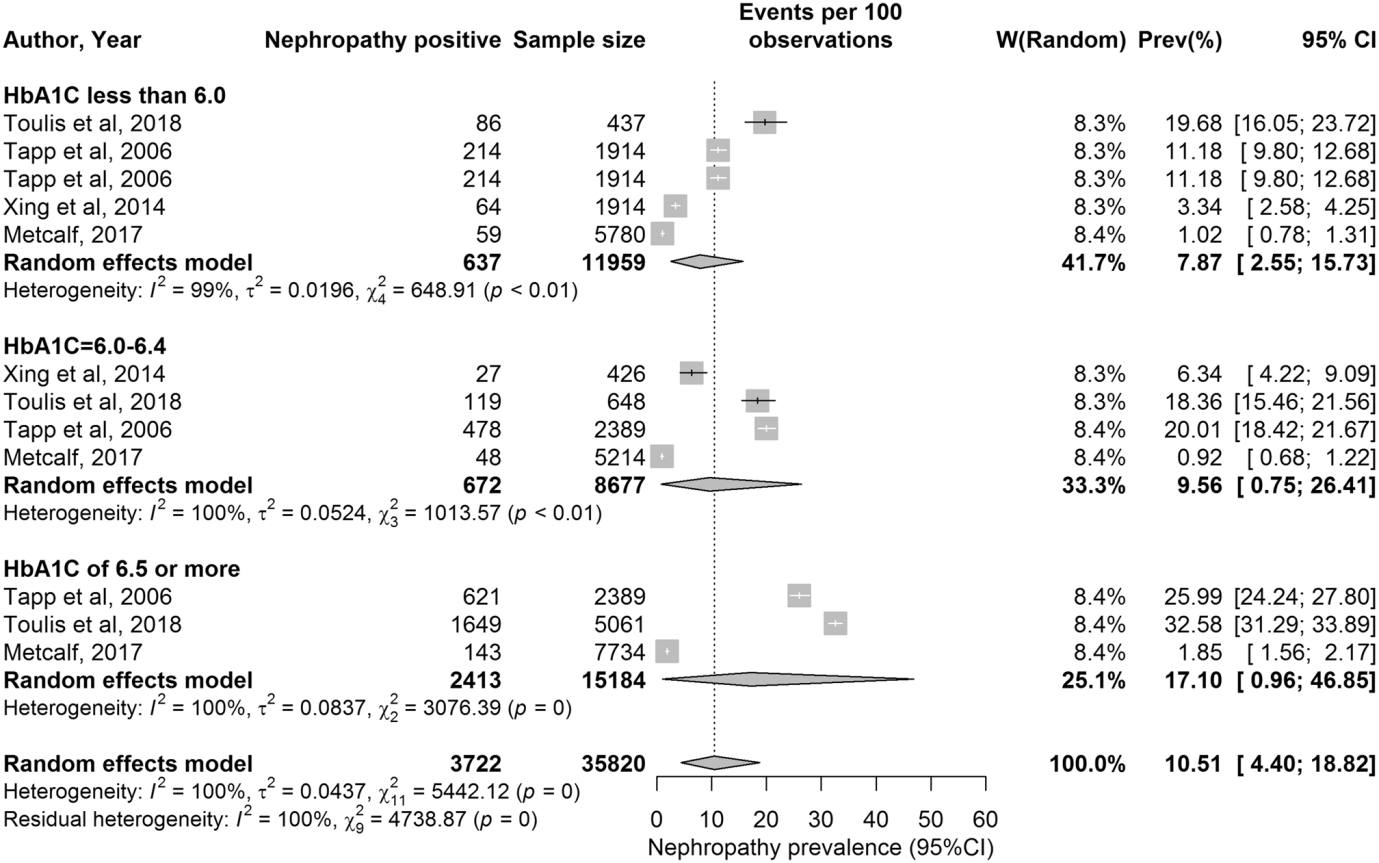
Forest plot showing results of the meta-analysis for nephropathy prevalence. Nephropathy prevalence stratified by HbA1c levels

The forest plot for nephropathy is shown in Fig. 2. The median prevalence of nephropathy was assessed at 11.2% (range: 0.9–32.6%). However, there was also variability based on HbA1c value, with a median of 7.3% (range: 1.0–19.7%) for HbA1c < 6.0% (< 42 mmol/mol), 12.4% (range: 0.9–18.5%) for HbA1c 6.0–6.4% (42–47 mmol/mol) and 26.0% (range: 1.9–32.6%) for HbA1c ≥ 6.5% (≥ 48 mmol/mol) (Table 4).

**Fig. 3:**
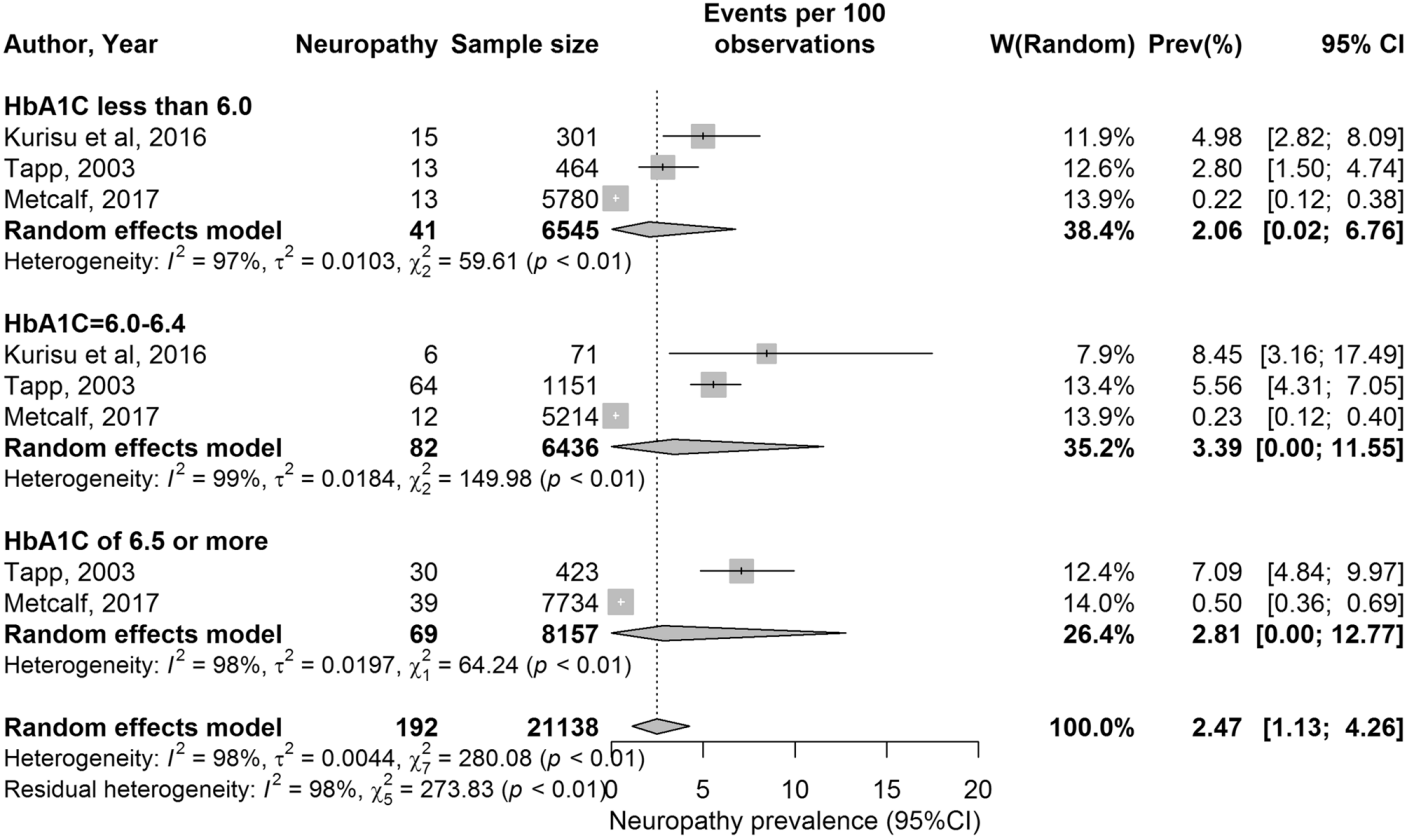
Forest plot showing results of the meta-analysis for neuropathy prevalence. Neuropathy prevalence stratified by HbA1c levels

The forest plot for neuropathy is shown in Fig. 3. The median prevalence for neuropathy was 3.9% (range: 0.2–8.5%). It was 2.8% (range: 0.2–5.0%) for HbA1c < 6.0% (< 42 mmol/mol), 5.6% (range: 0.2–8.5%) for HbA1c 6.0–6.4% (42–47 mmol/mol) and 3.8% (range: 0.2–8.5%) for HbA1c ≥ 6.5% (≥ 48 mmol/mol) (Table 4).

### Quality assessment

Supplementary Tables 3 and 4 show the results of the summarized and study-specific quality assessments. In sum, 72.7% (*n* = 16 out of 22) of retinopathy studies, 50.0% (*n* = 2 out of 4) of nephropathy studies and 66.7% (*n* = 2 out of 3) of neuropathy studies included at least 100 participants and therefore were considered as having higher precision.

Low risk of bias, assessed as consistency in measuring HbA1c across study participants, was found in 81.8% of studies assessing retinopathy, all studies assessing nephropathy, and 66.7% of studies assessing neuropathy. The majority of studies assessing retinopathy (95.5%) and all studies assessing nephropathy and neuropathy used probability-based sampling and hence were also classified as having low ROB on that quality domain.

Overall, studies reporting the prevalence of microvascular complications of T2DM were of acceptable quality: 81.8% of retinopathy studies, all nephropathy studies and 66.7% of neuropathy studies had low ROB on both quality domains. High ROB on both domains was found in only 4.5% of retinopathy studies and none of nephropathy or neuropathy studies.

### Meta-analysis results

The pooled mean prevalence was estimated at 4.1% (95% CI: 3.2–5.0%) for retinopathy, 10.5% (95% CI: 4.4–18.8%) for nephropathy and 2.5% (95% CI: 1.1–4.3%) for neuropathy (Table 4 and Figs. 1, 2, 3).

There was evidence for heterogeneity in prevalence estimates across all meta-analyses (Table 4). *P* value for Cochran’s *Q* statistic was almost always < 0.0001. *I*^2^ was > 90% indicating that most variability is due to true differences in prevalence across studies rather than chance.

#### HbA1c Level and risk of retinopathy

**Fig. 4:**
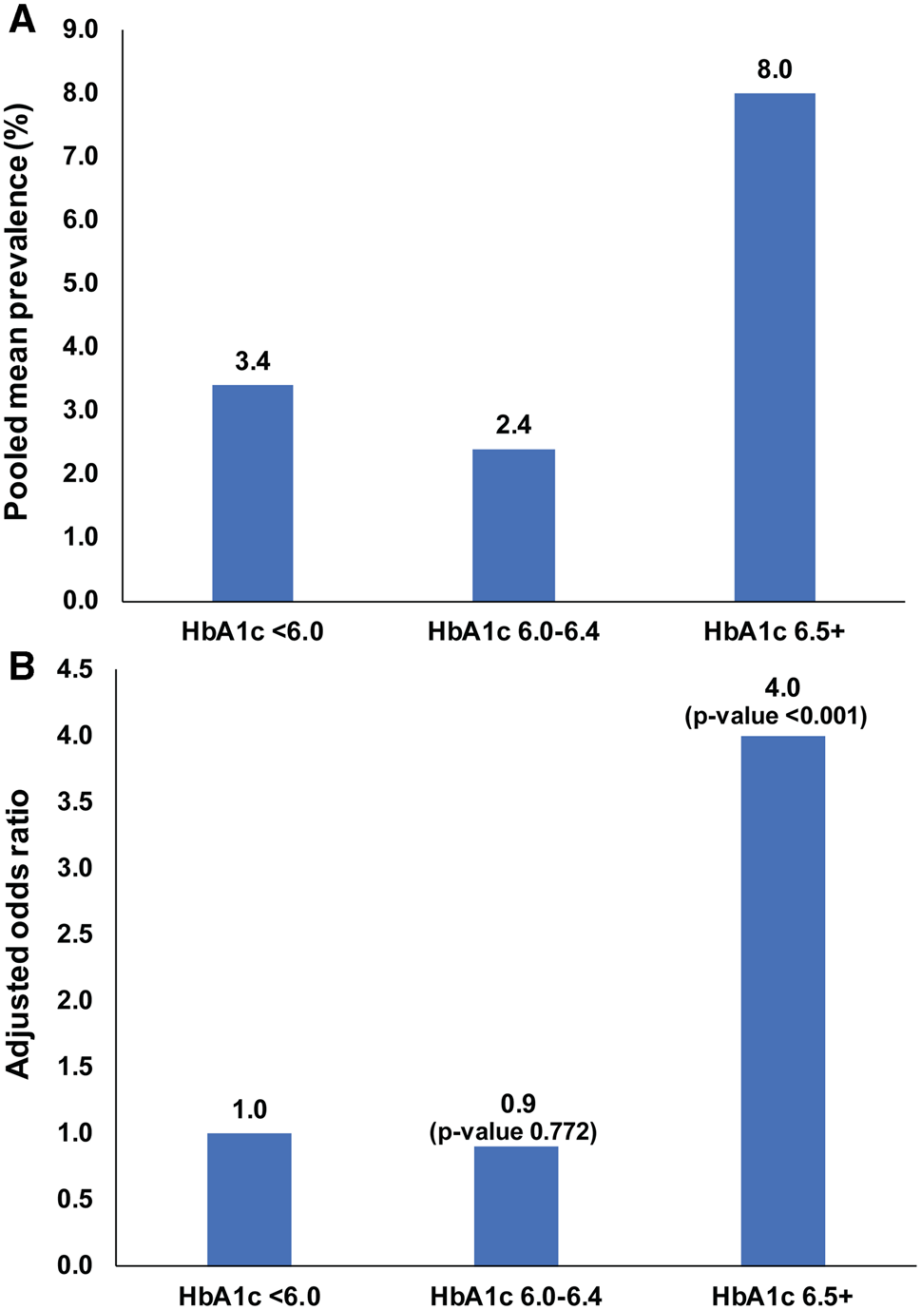
Pooled mean prevalence and adjusted odds ratio of retinopathy according to HbA1c. **a** Prevalence of retinopathy (%) stratified by HbA1c levels of < 6.0%, 6.1–6.4% and 6.5% or greater, showing marked increase in retinopathy prevalence in the 6.5% or greater group. **b** Adjusted odds ratio for retinopathy prevalence (for age, sex and ethnicity) stratified by HbA1c levels of < 6.0%, 6.1–6.4% and 6.5% or greater, showing marked increase in the odds ratio in the 6.5% or greater group

**Fig. 5:**
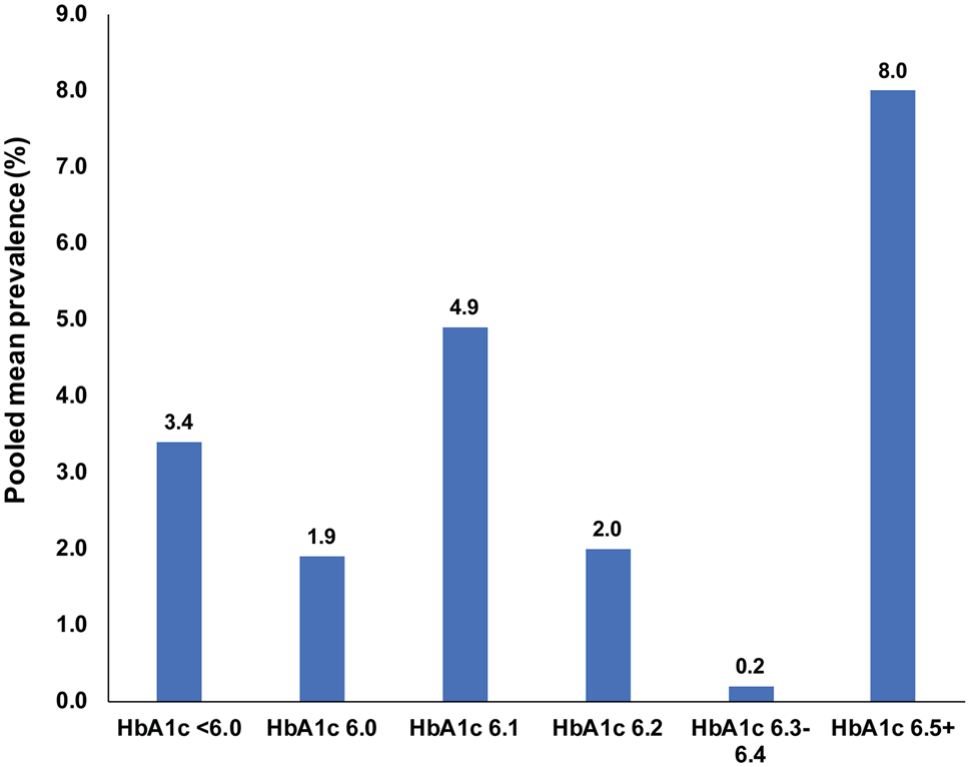
Pooled mean prevalence of retinopathy stratified in 0.1% increments of HbA1c. The prevalence of retinopathy stratified in 0.1% increments showed no trend of increasing retinopathy for HbA1c ranging from 6.0 to 6.4%, but a marked increase in prevalence at HbA1c of 6.5% and greater

For retinopathy, when the prevalence of complications was stratified according to HbA1c, using categories of < 6.0% (< 42 mmol/mol), 6.0–6.4% (42–46 mmol/mol) and ≥ 6.5% (≥ 48 mmol/mol), there was a distinct increase in retinopathy prevalence from a pooled mean of 3.41% (95% CI: 1.84–5.42) in the < 6.0% (< 42 mmol/mol) stratum and a pooled mean of 2.40% (95% CI: 1.65–3.29) in the 6–6.4% 42–47 mmol/mol) stratum to a pooled mean of 7.97% (95% CI: 5.70–10.57) in the ≥ 6.5% (≥ 48 mmol/mol) stratum. The J-shaped curve describing this association can be found in Fig. 4. The prevalence of retinopathy stratified in 0.1% increments is shown in Fig. 5. The latter analysis showed no trend of increasing retinopathy for HbA1c ranging from 6.0 to 6.4%, but a marked increase in prevalence at HbA1c of 6.5% and greater.

#### HbA1c Level and risk of nephropathy

The albumin/creatinine ratio (expressed as mg albumin: mmol creatinine; ACR) is a sensitive indicator of kidney disease, and patients are classified according to the 2012 guidelines developed by the Kidney Disease: Improving Global Outcomes organization (KDIGO) [52] as: normal < 3 mg/mmol (A1), moderate 3–30 mg/mmol (A2), severe > 30 mg/mmol (A3; nephropathy). Notably, for nephropathy, the prevalence of this complication overall tended to be higher than that for retinopathy. Again, there was a distinct increase in nephropathy prevalence from a pooled mean of 7.12% (95% CI: 1.68–15.85) in the < 6.0% (< 42 mmol/mol) stratum and a pooled mean of 9.56% (95% CI: 0.75–26.41) in the 6–6.4% (42–47 mmol/mol) stratum to a pooled mean of 17.10% (95% CI: 0.96–46.85) in the ≥ 6.5% (≥ 48 mmol/mol) stratum.

#### HbA1c Level and risk of neuropathy

Diabetic neuropathy, defined as the signs and symptoms of neuropathy wherein diabetes is the underlying cause, most frequently manifests as a distal, symmetric deficit [53]. For neuropathy, no trend in the pooled mean was seen when stratified according to HbA1c: < 6.0% (< 42 mmol/mol) (pooled mean of 2.06%; 95% CI: 0.02–6.76), 6–6.4% (42–47 mmol/mol) (pooled mean 3.39%; 95% CI: 0.00–11.55) and ≥ 6.5% (≥ 48 mmol/mol) (pooled mean 2.81%; 95% CI: 0.00–12.77). This may, however, be due to the paucity of studies available.

#### Subgroup Analysis

As a higher prevalence of complications may be anticipated with increasing age, the meta-analysis data of studies reporting retinopathy were stratified by age using both two strata (age ≤ 55 years and > 55 years) and three strata (age ≤ 55 years, age 55–59 years and > 60 years) with levels of HbA1c (Table 5). As anticipated, the pooled means did tend to increase with age, though this trend did not differ substantially from the original analysis.

**Table 5:** Results of meta-analyses of studies reporting retinopathy in patients with different HbA1C stratified by age

	Studies	Sample		Prevalence (%)				Heterogeneity measures	
	Total *N*	Tested	Number positive	Median	Range	Pooled mean	95% CI	*Q*^a^ (*p* value)	I^2b^ (%, 95% CI)
Retinopathy									
Age ≤ 55 years									
HbA1C < 6.0	4	9944	160	2.10	0.10–9.70	2.38	0.63–5.16	118.4 (*p* < 0.0001)	97.5 (95.6–98.5)
HbA1C = 6–6.4	15	30,299	274	0.30	0.02–11.60	1.17	0.52–2.07	506.9 (*p* < 0.0001)	97.2 (96.4–97.9)
HbA1C ≥ 6.5	11	12,771	574	5.00	0.40–19.10	6.70	3.64–10.57	459.6 (*p* < 0.0001)	97.8 (97.1–98.4)
Age > 55 years									
HbA1C < 6.0	14	53,988	1,548	3.65	0.0–15.90	3.73	1.75–6.37	2166.5 (*p* < 0.0001)	99.4 (99.3–99.5)
HbA1C = 6–6.4	17	61,623	1,415	3.85	0.62–14.80	3.80	2.59–5.23	1001.6 (*p* < 0.0001)	98.4 (98.0–98.7)
HbA1C ≥ 6.5	13	43,130	2,352	6.44	1.57–48.30	9.16	5.83–13.14	1715.9 (*p* < 0.0001)	99.3 (99.2–99.4)
Age < 55 years									
HbA1C < 6.0	4	9944	160	2.10	0.10–9.70	2.38	0.63–5.16	118.4 (*p* < 0.0001)	97.5 (95.6–98.5)
HbA1C = 6–6.4	15	30,299	274	0.30	0.02–11.60	1.17	0.52–2.07	506.9 (*p* < 0.0001)	97.2 (96.4–97.9)
HbA1C ≥ 6.5	11	12,771	574	5.00	0.40–19.10	6.70	3.64–10.57	459.6 (*p* < 0.0001)	97.8 (97.1–98.4)
Age 55–59 years									
HbA1C < 6.0	5	43,074	523	1.45	0.60–6.0	2.29	1.12–3.85	305.3 (*p* < 0.0001)	98.7 (98.1–99.1)
HbA1C = 6–6.4	11	57,555	1,209	3.85	0.99–10.00	3.84	2.47–5.50	788.3 (*p* < 0.0001)	98.7 (98.4–99.0)
HbA1C ≥ 6.5	10	40,452	2,186	7.40	1.70–22.20	8.97	5.24–13.57	1653.2 (*p* < 0.0001)	99.5 (99.3–99.6)
Age ≥ 60 years									
HbA1C < 6.0	9	10,914	1,025	5.00	0.00–15.90	4.69	1.58–9.26	637.5 (*p* < 0.0001)	98.7 (98.4–99.0)
HbA1C = 6–6.4	6	4,068	206	3.06	0.62–14.80	3.72	1.22–7.44	119.1 (*p* < 0.0001)	95.8 (93.1–97.5)
HbA1C ≥ 6.5	3	2678	166	6.44	1.57–48.30	10.84	2.65–23.19	53.7 (*p* < 0.0001)	96.3 (92.1–98.2)

^a^*Q*: the Cochran’s *Q* statistic is a measure assessing the existence of heterogeneity in effect size (here, prevalence) across studies

^b^*I*^2^: a measure assessing the magnitude of between-study variation that is due to differences in effect size (here, prevalence) across studies rather than chance

Certain ethnic populations are also known to develop diabetes complications more frequently, notably African-Americans [46]. A sensitivity analysis was therefore performed on the results of the meta-analysis for studies reporting retinopathy separately for African-Americans and non-Black populations (Table 6). The results showed the same upward trend for retinopathy with increasing levels of HbA1c in both the Black and non-Black populations, though the African-Americans populations had a notably higher prevalence of retinopathy at every level of HbA1c; the prevalence of retinopathy in non-African-Americans in those studies did not differ to the overall non-African American prevalence.

**Table 6:** Sensitivity analysis showing results of meta-analyses of studies reporting retinopathy in patients with different HbA1C excluding African-American patients

	Studies	Sample		Prevalence (%)				Heterogeneity measures	
	Total *N*	Tested	Number positive	Median	Range	Pooled mean	95% CI	*Q*^a^ (*p* value)	*I*^2^^b^ (%, 95% CI)
Retinopathy									
Non-African-American									
HbA1C < 6.0	16	63,483	1660	1.88	0.00–15.90	2.79	1.34–4.72	2232.1 (*p* < 0.0001)	99.3 (99.2–99.4)
HbA1C = 6–6.4	29	90,744	1548	1.07	0.02–10.00	1.87	1.22–2.64	1599.7 (*p* < 0.0001)	98.2 (97.9–98.5)
HbA1C ≥ 6.5	21	54,997	2748	5.14	0.40–22.20	6.67	4.59–9.10	1956.6 (*p* < 0.0001)	99.0 (98.8–99.1)
African-American									
HbA1C < 6.0	2	449	48	12.05	9.70–14.40	11.14	6.98–16.09	1.8 (*p* = 0.1793)	44.6 (–)
HbA1C = 6–6.4	3	1178	141	11.6	10.90–14.80	11.89	10.09–13.81	1.7 (*p* = 0.4192)	0.0 (0.0–88.0)
HbA1C ≥ 6.5	3	904	178	19.10	14.80–48.30	23.94	12.08–38.19	12.6 (*p* = 0.0018)	84.1 (52.4–94.7)

^a^*Q*: the Cochran’s *Q* statistic is a measure assessing the existence of heterogeneity in effect size (here, prevalence) across studies

^b^*I*^2^: a measure assessing the magnitude of between-study variation that is due to differences in effect size (here, prevalence) across studies rather than chance

There were insufficient studies for both nephropathy and neuropathy to perform subgroup analyses.

#### Meta-regression results

Only retinopathy prevalence had a sufficient number of studies to warrant conduct of meta-regression analysis (Table 7).

**Table 7:** Results of univariable meta-regression analyses for the prevalence of retinopathy by key factors

Predictors		Studies/strata	Samples	Univariable analysis			Variance explained	Multivariable analysis^a^		
		Total *N*	Total *n*	OR (95% CI)	*p* value^b^	*F* test *p* value	*R*^2^ (%)	OR (95% CI)	*p* value^a^	*F* test *p* value
HbA1C	< 6.0	18	63,932	1.00		< 0.001	16.9	1.00		< 0.001
	6.0–6.4	32	91,922	0.64 (0.27–1.53)	0.313			0.90 (0.43–1.86)	0.772	
	6.5 +	24	55,901	3.24 (1.29–8.15)	0.013			3.96 (1.85–8.50)	0.001	
Age	< = 55 years	29	47,180	1.00	0.017	0.017	6.4	1.00	< 0.001	< 0.001
	> 55 years	45	164,575	2.50 (1.19–5.28)				3.33 (1.83–6.04)		
Race	White	66	209,224	1.00		< 0.001	16.2	1.00	< 0.001	< 0.001
	Black	8	2531	8.71 (2.87–26.43)	< 0.001			10.95 (4.36–27.50)		

^a^The multivariable model explained 44.3% of the variation in retinopathy prevalence

^b^Strength of evidence for an association with prevalence was deemed “good” at *p* value ≤ 0.1 and “strong” at *p* value ≤ 0.05

The univariable analysis showed an association with prevalence for HbA1c levels, age and race; these were therefore included in the multivariable model. Here, HbA1c stratification showed a marked and significant increase of retinopathy prevalence at ≥ 6.5% with an AOR of 4.0 (95% CI: 1.9–8.5; *p* < 0.001) after controlling for the confounding effect of age and race. The model also showed that individuals > 55 years of age had threefold higher odds for retinopathy than younger individuals (AOR: 3.3; 95% CI: 1.8–6.0; *p* < 0.001). African-Americans also had significantly higher odds for retinopathy prevalence than those of other race (AOR: 11.0; 95% CI: 4.4–27.5; *p* < 0.001). This model explained 44.8% of the variation in retinopathy prevalence.

## Discussion

The meta-analysis relating stratified HbA1c to the prevalence of retinopathy, nephropathy and neuropathy showed clearly that for moderate retinopathy, the recent data are in accord with a HbA1c diagnostic cutoff of 6.5% (48 mmol/mol and above) for T2DM, with the inflection point for the increase in diabetic retinopathy prevalence being at 6.5% (48 mmol/mol), as also shown by the stratified analysis using the *J*-shaped curve. Pooled mean retinopathy prevalence by 0.1% HbA1c changes showed no obvious signal of a rising retinopathy prevalence for HbA1c 6.0 to 6.4%, with the inflection point being at 6.5% or greater. This being said, there were too few studies assessing retinopathy prevalence at HbA1c increments of 6.3% and 6.4% for evidence to be conclusive. The latter analysis also showed considerable retinopathy prevalence at below 6.0% (42 mmol/mol) HbA1c. It should be also emphasized that the stratification of HbA1c and retinopathy was based on the detection of moderate retinopathy and it is unknown whether the diagnostic cutpoint for T2DM may be altered by prevalence studies on minimal diabetic retinopathy. These results differ from that reported in a previous meta-analysis that concluded that HbA1c thresholds could not be identified from the studies on microvascular complications, though the threshold of HbA1c of 6.5% was strong for severe retinopathy [13].

The heterogeneity in the retinopathy data was controlled for in the meta-regression and showed, in both the univariate and multivariate analyses, the increased odds ratio of 3.2 and 4.05, respectively, with an HbA1c of 6.5% or greater; however, as noted the stratified HbA1c data revealed a *J*-shaped curve showing accountable retinopathy prevalence at below 6.0%. The meta-regression also revealed the increase in retinopathy prevalence with those aged over 55 years, with an OR of 3.23 and a striking increase in retinopathy prevalence for African-Americans with an OR of 10.73 on multivariate analysis. While this is in accord with the previous data [3, 9–11], retinopathy clearly occurs earlier than the cutoff of 6.5%. The magnitude of the association of retinopathy with race found here may not be representative as only 8 studies were available among African-Americans, though it raises the question whether a different diagnostic cutoff for T2DM for African-Americans aged greater than 55 years may be appropriate, as they would appear to be the group at greatest risk [54]. It should also be emphasized that the stratification of HbA1c and retinopathy was based on the detection of moderate retinopathy and it is unknown whether the diagnostic cutpoint for T2DM may be altered by prevalence studies on minimal diabetic retinopathy. Thus, the T2DM diagnostic cutpoint for HbA1c may not be a universal “one size fits all,” but may need to be stratified according to age and race.

The main limitations of the 22 studies of retinopathy included in the meta-analysis were that retinal photography was not standardized and that the degree of retinopathy was often reported as moderate or poorly specified [27, 38]. As different studies have measured the complications in different ways, this may have affected the outcome and we may therefore have underestimated the prevalence of the diabetic complications. There were few studies focusing specifically on the onset of minimal diabetes retinopathy. In addition, minimal retinopathy may be difficult to differentiate between diabetes and non-diabetes-related retinopathy and therefore only moderate diabetes changes were identified in all studies analyzed. This could suggest that with a standardized procedure specifically identifying minimal diabetic retinopathy, that the inflection point for the onset of retinopathy would be a HbA1c lower than 6.5% (48 mmol/mol), but this is clearly speculative as there is insufficient evidence to support this at present.

Four studies were evaluated which considered the onset of nephropathy, the data showing that the HbA1c cutoff of 6.5% (48 mmol/mol) was appropriate with a significant increase in the urine albumin/creatinine ratio. Again, it is evident that there were significant numbers of subjects with albuminuria in the range of “prediabetes” between 6.0–6.4% (42–47 mmol/mol); however, the main limitations were the small number of studies, heterogeneity of the population and lack of division of the HbA1c level into 0.1% ranges that prevented a more stringent determination. In addition, all of the studies were cross sectional with a need for longitudinal studies to be performed.

There were only three studies with sufficient data that could be evaluated for the onset of neuropathy, and these were inconclusive with no inflection point seen for the HbA1c cutoff, with a prevalence of neuropathy at an HbA1c of 6.0–6.4 (42–46 mmol/mol) being the same as that of 6.5% (48 mmol/mol) and above. The main limitations here include the limited amount of data in too few studies, heterogeneity of the population and poor division of the HbA1c range into 0.1% ranges; however, given the limited data, it is unlikely that division of HbA1c into 0.1% ranges would have allowed a more stringent determination.

## Conclusions

In conclusion, based on the strongest data of moderate retinopathy, this systematic review and meta-analysis is in accord that the HbA1c diagnostic cutpoint of 6.5% (48 mmol/mol) is highly specific for diagnosing T2DM, though the increased prevalence of retinopathy in those aged 55 years or greater and in African-Americans may suggest a lower threshold is appropriate for those groups. However, this analysis highlights that diabetic microvascular complications commonly occur at lower HbA1c values, and the sensitivity of diagnosing T2DM may improve with a lower diagnostic cutpoint. There is a need for studies that standardize the definition of minimal diabetic retinopathy with more sensitive measures of nephropathy and neuropathy to determine if a lower threshold is appropriate.

## Electronic supplementary material

Below is the link to the electronic supplementary material.

PRISMA Flow Diagram (PDF 215 kb)

PRISMA checklist (DOCX 20 kb)

Literature search (DOCX 32 kb)

Summary of precision and risk of bias (ROB) assessment. Summary of precision and risk of bias (ROB) assessment for studies reporting the prevalence of microvascular complications associated with type 2 diabetes mellitus (DOCX 17 kb)

Study-specific quality assessments (DOCX 19 kb)

## Data Availability

All the data for this study will be made available upon reasonable request to the corresponding author.

## References

[1] DF Diabetes Atlas (2017); 8th:ttp://www.diabetesatlas.org

[2] Targets G (2019). Standards of medical care in diabetes-2019. Diabetes Care.

[3] Report of the expert committee on the diagnosis and classification of diabetes mellitus (1997) Diabetes Care, vol 20(7), pp1183–1197 10.2337/diacare.20.7.1183 9203460

[4] Genuth S, Alberti KG, Bennett P (2003). Follow-up report on the diagnosis of diabetes mellitus. Diabetes Care.

[5] Zhang X, Gregg EW, Williamson DF (2010). A1C level and future risk of diabetes: a systematic review. Diabetes Care.

[6] Selvin E, Ning Y, Steffes MW (2011). Glycated hemoglobin and the risk of kidney disease and retinopathy in adults with and without diabetes. Diabetes.

[7] Ackermann RT, Cheng YJ, Williamson DF, Gregg EW (2011). Identifying adults at high risk for diabetes and cardiovascular disease using hemoglobin A1c national health and nutrition examination survey 2005–2006. Am J Prev Med.

[8] Gillett MJ (2009). International expert committee report on the role of the A1c assay in the diagnosis of diabetes: diabetes care 2009; 32(7): 1327–1334. Clin Biochem Rev.

[9] Colagiuri S, Lee CM, Wong TY, Balkau B, Shaw JE, Borch-Johnsen K (2011). Glycemic thresholds for diabetes-specific retinopathy: implications for diagnostic criteria for diabetes. Diabetes Care.

[10] McCance DR, Hanson RL, Charles MA (1994). Comparison of tests for glycated haemoglobin and fasting and two hour plasma glucose concentrations as diagnostic methods for diabetes. BMJ (Clin Res Ed).

[11] Engelgau MM, Thompson TJ, Herman WH (1997). Comparison of fasting and 2-hour glucose and HbA1c levels for diagnosing diabetes. Diagnostic criteria and performance revisited. Diabetes Care.

[12] Cowie CC, Rust KF, Byrd-Holt DD (2010). Prevalence of diabetes and high risk for diabetes using A1C criteria in the US population in 1988–2006. Diabetes Care.

[13] Kowall B, Rathmann W (2013). HbA1c for diagnosis of type 2 diabetes. Is there an optimal cut point to assess high risk of diabetes complications, and how well does the 6.5% cutoff perform?. Diabetes, Metab Syndr Obes Targets Ther.

[14] Higgins JP, Green S (2011). Cochrane handbook for systematic reviews of interventions.

[15] Moher D, Liberati A, Tetzlaff J, Altman DG, Group P (2009). Preferred reporting items for systematic reviews and meta-analyses: the PRISMA statement. J Clin Epidemiol.

[16] McGowan J, Sampson M, Salzwedel DM, Cogo E, Foerster V, Lefebvre C (2016). PRESS peer review of electronic search strategies: 2015 guideline statement. J Clin Epidemiol.

[17] Higgins JPT, Green S (2008). Cochrane Collaboration Cochrane handbook for systematic reviews of interventions.

[18] Freeman MF, Tukey JW (1950). Transformations related to the angular and the square root. Ann Math Stat.

[19] Miller JJ (1978). The inverse of the freeman—Tukey double arcsine transformation. Am Stat.

[20] Barendregt JJ, Doi SA, Lee YY, Norman RE, Vos T (2013). Meta-analysis of prevalence. J Epidemiol Commun Health.

[21] DerSimonian R, Laird N (1986). Meta-analysis in clinical trials. Control Clin Trials.

[22] Borenstein M (2009). Introduction to meta-analysis.

[23] Higgins JP, Thompson SG (2002). Quantifying heterogeneity in a meta-analysis. Stat Med.

[24] R core team (2017) R: a language and environment for statistical computing. Vienna, Austria: R Foundation for Statistical Computing

[25] StataCorp (2015) Stata Statistical Software: Release 14. College Station, TX: StataCorp LP. 2015

[26] Harbord RM, Higgins JPT (2008). Meta-regression in Stata. Stata J.

[27] Ito C, Maeda R, Ishida S, Harada H, Inoue N, Sasaki H (2000). Importance of OGTT for diagnosing diabetes mellitus based on prevalence and incidence of retinopathy. Diabetes Res Clin Pract.

[28] Tapp RJ, Zimmet PZ, Harper CA (2006). Diagnostic thresholds for diabetes: the association of retinopathy and albuminuria with glycaemia. Diabetes Res Clin Pract.

[29] Samadi Aidenloo N, Mehdizadeh A, Valizadeh N, Abbaszadeh M, Qarequran S, Khalkhali H (2016). Optimal glycemic and hemoglobin A1c thresholds for diagnosing diabetes based on prevalence of retinopathy in an Iranian population. Irani Red Crescent Medl J.

[30] Almdal TP, Handlos LN, Valerius M (2014). Glycaemic threshold for diabetes-specific retinopathy among individuals from Saudi Arabia, Algeria and Portugal. Diabetes Res Clin Pract.

[31] Bertelsen G, Peto T, Lindekleiv H (2014). Sex differences in risk factors for retinopathy in non-diabetic men and women: the tromso eye study. Acta Ophthalmol.

[32] Bower JK, Brancati FL, Selvin E (2013). No ethnic differences in the association of glycated hemoglobin with retinopathy: the national health and nutrition examination survey 2005–2008. Diabetes Care.

[33] Cheng YJ, Gregg EW, Geiss LS (2009). Association of A1c and fasting plasma glucose levels with diabetic retinopathy prevalence in the US population: implications for diabetes diagnostic thresholds. Diabetes Care.

[34] Cho NH, Kim TH, Woo SJ (2013). Optimal HbA1c cutoff for detecting diabetic retinopathy. Acta Diabetol.

[35] Fukushima S, Nakagami T, Suto C, Hirose A, Uchigata Y (2013). Prevalence of retinopathy and its risk factors in a Japanese population. J Diabetes Invest.

[36] Park YM, Ko SH, Lee JM (2014). Glycaemic and haemoglobin A1c thresholds for detecting diabetic retinopathy: the fifth Korea national health and nutrition examination survey (2011). Diabetes Res Clin Pract.

[37] Lamparter J, Raum P, Pfeiffer N (2014). Prevalence and associations of diabetic retinopathy in a large cohort of prediabetic subjects: the Gutenberg health study. J Diabetes Complicat.

[38] Metcalf PA, Kyle C, Kenealy T, Jackson RT (2017). HbA1c in relation to incident diabetes and diabetes-related complications in non-diabetic adults at baseline. J Diabetes Complicat.

[39] Nakagami T, Takahashi K, Suto C (2017). Diabetes diagnostic thresholds of the glycated hemoglobin A1c and fasting plasma glucose levels considering the 5-year incidence of retinopathy. Diabetes Res Clin Pract.

[40] Okosun IS, Turbow S, McJenkin K, Monique Davis-Smith Y, Seale JP (2016). Diagnostic performance of glycated hemoglobin for diabetic retinopathy in non-diabetic older overweight/obese African-Americans. Diabetes Res Clin Pract.

[41] Pang C, Jia L, Jiang S (2012). Determination of diabetic retinopathy prevalence and associated risk factors in Chinese diabetic and pre-diabetic subjects: Shanghai diabetic complications study. Diabetes/Metabol Res Rev.

[42] Sabanayagam C, Khoo EY, Lye WK (2015). Diagnosis of diabetes mellitus using HbA1c in Asians: relationship between HbA1c and retinopathy in a multiethnic Asian population. J Clin Endocrinol Metabol.

[43] Sabanayagam C, Liew G, Tai ES (2009). Relationship between glycated haemoglobin and microvascular complications: is there a natural cut-off point for the diagnosis of diabetes?. Diabetologia.

[44] Tsugawa Y, Takahashi O, Meigs JB (2012). New diabetes diagnostic threshold of hemoglobin A(1c) and the 3-year incidence of retinopathy. Diabetes.

[45] Massin P, Lange C, Tichet J (2011). Hemoglobin A1c and fasting plasma glucose levels as predictors of retinopathy at 10 years: the French DESIR study. Arch Ophthalmol.

[46] Tsugawa Y, Mukamal KJ, Davis RB, Taylor WC, Wee CC (2012). Should the hemoglobin A1c diagnostic cutoff differ between blacks and whites? A cross-sectional study. Ann Intern Med.

[47] Toulis KA, Jiang CQ, Hemming K (2018). Glycated hemoglobin, albuminuria and surrogate markers of macrovascular disease in adults without diabetes: the Guangzhou Biobank cohort study, cardiovascular disease subcohort. Can J Diabetes.

[48] Xing FY, Neeland IJ, Gore MO (2014). Association of prediabetes by fasting glucose and/or haemoglobin A1c levels with subclinical atherosclerosis and impaired renal function: observations from the Dallas Heart Study. Diabetes Vasc Dis Res.

[49] Tapp RJ, Shaw JE, de Courten MP, Dunstan DW, Welborn TA, Zimmet PZ (2003). Foot complications in Type 2 diabetes: an Australian population-based study. Diabetic Med.

[50] Kurisu SO KI, Sasaki H, Tanaka H, Yamaneki M, Nakanishi I, Furuta H, et al. (2016) Polyneuropathy or neuropathic pain did not increase at prediabetic stage in Japanese population. J Diabetes Invest

[51] Solomon SD, Chew E, Duh EJ (2017). Diabetic retinopathy: a position statement by the American diabetes association. Diabetes Care.

[52] Stevens PE, Levin A (2013). Kidney disease: improving global outcomes chronic kidney disease guideline development Work group M. Evaluation and management of chronic kidney disease: synopsis of the kidney disease: improving global outcomesclinical practice guideline. Ann Intern Med.

[53] Bansal V, Kalita J, Misra UK (2006). Diabetic neuropathy. Postgrad Med J.

[54] Chatterjee R, Maruthur NM, Edelman D (2015). Novel risk factors for type 2 diabetes in African–Americans. Curr DiabRep.

