# Supplementary material for: Diagnosing type 2 diabetes using Hemoglobin A1c: a systematic review and meta-analysis of the diagnostic cutpoint based on microvascular complications
Source: Acta Diabetol. 2020 Nov 3;58(3):279–300. doi: 10.1007/s00592-020-01606-5 (PMC7907031; doi:10.1007/s00592-020-01606-5)
Supplement: Supplementary file 1 — PRISMA Flow Diagram (PDF 215 kb) [file 592_2020_1606_MOESM1_ESM.pdf]

PRISMA 2009 Flow Diagram

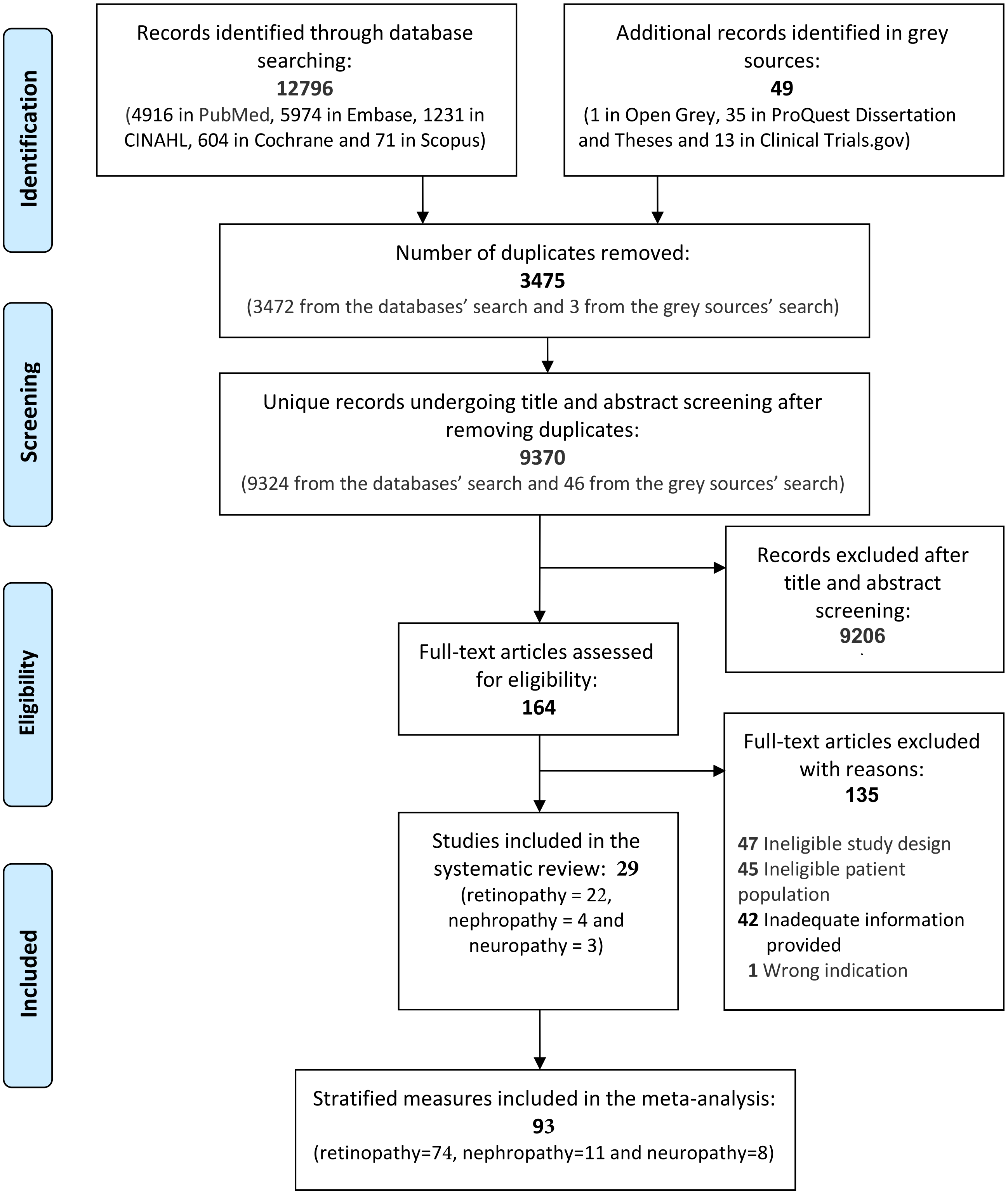

From: Moher D, Liberati A, Tetzlaff J, Altman DG, The PRISMA Group (2009). *P*referred *R*eporting *I*tems for *S*ystematic Reviews and *M*eta-*A*nalyses: The PRISMA Statement. PLoS Med 6(7): e1000097. doi:10.1371/journal.pmed1000097

For more information, visit www.prisma-statement.org.
