# Supplementary material for: Diagnosing type 2 diabetes using Hemoglobin A1c: a systematic review and meta-analysis of the diagnostic cutpoint based on microvascular complications
Source: Acta Diabetol. 2020 Nov 3;58(3):279–300. doi: 10.1007/s00592-020-01606-5 (PMC7907031; doi:10.1007/s00592-020-01606-5)
Supplement: Supplementary file 3 — Literature search (DOCX 32 kb) [file 592_2020_1606_MOESM3_ESM.docx]

**Supplementary Table 2**

**Literature search**

**Academic databases**

| **Source and date** | **Search string** | **Result** | **Notes** |
| --- | --- | --- | --- |
| **Source:**  PubMed  **Search date:**  2019-03-12 | (("Hb A1c"[Title/Abstract] OR “A1C” [Title/Abstract] OR “GHb”[Title/Abstract] OR “HbA1c”[Title/Abstract] OR “HbA1”[Title/Abstract] OR “Hb A1”[Title/Abstract] OR ”hemoglobin A1c”[Title/Abstract] OR ”hemoglobin A1c”[Title/Abstract] OR ”haemoglobin A1c”[Title/Abstract] OR ”haemoglobin A1c”[Title/Abstract] OR “glycohemoglobin”[Title/Abstract] OR “glyco hemoglobin”[Title/Abstract] OR “glycohemoglobins”[Title/Abstract] OR “glyco hemoglobins”[Title/Abstract] OR “glycohaemoglobin”[Title/Abstract] OR “glyco haemoglobin”[Title/Abstract] OR “glycohaemoglobins”[Title/Abstract] OR “glyco haemoglobins”[Title/Abstract] OR “hemoglobin A(1)”[Title/Abstract] OR “haemoglobin A(1)”[Title/Abstract] OR “glycosylated hemoglobin”[Title/Abstract] OR “glycosylated hemoglobins”[Title/Abstract] OR “glycosylated haemoglobin”[Title/Abstract] OR “glycosylated haemoglobins”[Title/Abstract] OR “glycated hemoglobins”[Title/Abstract] OR “glycated hemoglobin” [Title/Abstract] OR “glycated haemoglobins”[Title/Abstract] OR “glycated haemoglobin”[Title/Abstract] OR “Glycated Hemoglobin A"[Mesh]) AND ("Diabetes Mellitus"[Mesh] OR “T2D”[Title/Abstract] OR diabet*[Title/Abstract] OR “T2DM”[Title/Abstract] OR “NIDDM”[Title/Abstract] OR “MODY”[Title/Abstract] OR “IDDM”[Title/Abstract] OR prediabet*[Title/Abstract] OR "pre diabetic" [Title/Abstract] OR "pre diabetes" [Title/Abstract]) AND ("Diabetic Retinopathy"[Mesh] OR retinopathy[Title/Abstract] OR "Retinal Diseases"[Mesh:NoExp] OR "retinal disease"[Title/Abstract] OR "retinal diseases"[Title/Abstract] OR “retinal vascular disease”[Title/Abstract] OR “retinal vascular diseases”[Title/Abstract] OR retinopathies[Title/Abstract] OR neuropathy[Title/Abstract] OR neuropathies[Title/Abstract] OR "Diabetic Neuropathies"[Mesh] OR nephropathies[Title/Abstract] OR nephropathy[Title/Abstract] OR "Diabetic Nephropathies"[Mesh]) AND (diagnos*[Title/Abstract] OR "Diagnosis"[Mesh] OR threshold*[Title/Abstract] OR cutoff[Title/Abstract] OR "cut point"[Title/Abstract] OR cutpoint*[Title/Abstract] OR "cut points"[Title/Abstract] OR "cut off"[Title/Abstract])) | **4916** | All terms searched in the fields for title and abstract and in MeSH when available.  Publication year filter for 1990- applied  **MeSH inclusions:**  “Hb A1c“ and “Glycosylated Hemoglobin” is referred to "Glycated Hemoglobin A" in MeSH. Included in MeSH and automatically searched by “glycated hemoglobin” in TI/AB |
| **Embase**  (OVID)  **Search Date**  2019-03-11 | ((Hb A1c:ab,ti OR A1C:ab,ti OR GHb:ab,ti OR HbA1c:ab,ti OR HbA1:ab,ti OR Hb A1: ab,ti OR hemoglobin A1c:ab,ti OR hemoglobin A1:ab,ti OR haemoglobin A1c:ab,ti OR haemoglobin A1c:ab,ti OR glycohemoglobin:ab,ti OR glyco hemoglobin:ab,ti OR glycohemoglobins:ab,ti OR glyco hemoglobins:ab,ti OR glycohaemoglobin:ab,ti OR glyco haemoglobin:ab,ti OR glycohaemoglobins:ab,ti OR glyco haemoglobins:ab,ti OR hemoglobin A(1) :ab,ti OR haemoglobin A(1) :ab,ti OR glycosylated hemoglobin:ab,ti OR glycosylated hemoglobins:ab,ti OR glycosylated haemoglobin:ab,ti OR glycosylated haemoglobins:ab,ti OR glycated hemoglobins:ab,ti OR glycated hemoglobin:ab,ti OR glycated haemoglobins:ab,ti OR glycated haemoglobin:ab,ti OR ”Glycosylated Hemoglobin”/exp) AND (T2D:ab,ti OR diabet*:ab,ti OR T2DM:ab,ti OR NIDDM:ab,ti OR MODY:ab,ti OR IDDM:ab,ti OR prediabet*:ab,ti OR pre diabetic: ab,ti OR pre diabetes: ab,ti OR diabetes mellitus/exp) AND (Retinal Diseases/de OR Diabetic Nephropathies/de OR Diabetic Neuropathies /de OR Diabetic Retinopathy/de OR retinopathy:ab,ti OR retinal disease:ab,ti OR retinal diseases:ab,ti OR retinal vascular disease:ab,ti OR retinal vascular diseases:ab,ti OR retinopathies:ab,ti OR neuropathy:ab,ti OR neuropathies:ab,ti OR nephropathies:ab,ti OR nephropathy:ab,ti) AND (Diagnosis/exp OR diagnos*:ab,ti OR threshold*ab,ti OR cutoff:ab,ti OR cut point:ab,ti OR cutpoint*:ab,ti OR cut points:ab,ti OR cut off:ab,ti)) | **5974** | All terms searched in the fields for title and abstract (here marked with “:ab,ti”) and in MeSH (here marked with “/de or /exp”) when available  Publication year filter for 1990- applied  **MeSH/thesaurus variations compared to PubMed:**  “Glycated Hemoglobin A" is referred to “Glycosylated Hemoglobin” in Embase Emtree. Included. |
| **CINAHL**  **Search Date:**  2019-03-12 | ((TI ("Hb A1c" OR “A1C” OR “GHb” OR “HbA1c” OR “HbA1” OR “Hb A1” OR ”hemoglobin A1c”OR ”hemoglobin A1c” OR ”haemoglobin A1c” OR ”haemoglobin A1c” OR “glycohemoglobin” OR “glyco hemoglobin” OR “glycohemoglobins” OR “glyco hemoglobins” OR “glycohaemoglobin” OR “glyco haemoglobin” OR “glycohaemoglobins” OR “glyco haemoglobins” OR “hemoglobin A(1)” OR “haemoglobin A(1)” OR “glycosylated hemoglobin” OR “glycosylated hemoglobins” OR “glycosylated haemoglobin” OR “glycosylated haemoglobins” OR “glycated hemoglobins” OR “glycated hemoglobin” OR “glycated haemoglobins” OR “glycated haemoglobin”) OR AB ("Hb A1c" OR “A1C” OR “GHb” OR “HbA1c” OR “HbA1” OR “Hb A1” OR ”hemoglobin A1c”OR ”hemoglobin A1c” OR ”haemoglobin A1c” OR ”haemoglobin A1c” OR “glycohemoglobin” OR “glyco hemoglobin” OR “glycohemoglobins” OR “glyco hemoglobins” OR “glycohaemoglobin” OR “glyco haemoglobin” OR “glycohaemoglobins” OR “glyco haemoglobins” OR “hemoglobin A(1)” OR “haemoglobin A(1)” OR “glycosylated hemoglobin” OR “glycosylated hemoglobins” OR “glycosylated haemoglobin” OR “glycosylated haemoglobins” OR “glycated hemoglobins” OR “glycated hemoglobin” OR “glycated haemoglobins” OR “glycated haemoglobin”) OR (MH "Hemoglobin A, Glycosylated") AND (TI ( “T2D” OR diabet* OR “T2DM” OR “NIDDM” OR “MODY” OR “IDDM” OR Prediabet* OR "Pre diabetic" OR "Pre diabetes") OR AB (“T2D” OR diabet* OR “T2DM” OR “NIDDM” OR “MODY” OR “IDDM” OR prediabet* OR "pre diabetic" OR "pre diabetes") OR (MH "Diabetes Mellitus+") AND (TI (retinopathy OR "retinal disease" OR "retinal diseases" OR “retinal vascular disease” OR “retinal vascular diseases” OR retinopathies OR neuropathy OR neuropathies OR nephropathies OR nephropathy) OR AB ( retinopathy OR "retinal disease" OR "retinal diseases" OR “retinal vascular disease” OR “retinal vascular diseases” OR retinopathies OR neuropathy OR neuropathies OR nephropathies OR nephropathy) OR (MH “Diabetic Nephropathies”) OR (MH "Diabetic Neuropathies") OR (MH "Diabetic Retinopathy") OR (MH "Retinal Diseases") AND TI ( diagnos* OR threshold* OR cutoff OR "cut point" OR cutpoint*OR "cut points" OR "cut off") OR AB (diagnos* OR threshold* OR cutoff OR "cut point" OR cutpoint*OR "cut points" OR "cut off" OR (MH "Diagnosis+”)) | **1231** | All terms searched in the fields for title and abstract (here marked with “TI” and “AB”) and in MeSH (here marked with “MH”) when available  Publication year filter, 1990- applied  **MeSH/thesaurus variations compared to PubMed:**  "Glycated Hemoglobin A" is referred to “[Hemoglobin A, Glycosylated](javascript:XslPostBack('ctl00$ctl00$MainContentArea$MainContentArea$ctrlResults','meshDetail','index%7C1%24term%7CHemoglobin%20A%2C%20Glycosylated%24cmd%7CmeshDetail');)” in CINAHL’s Subject Headings. Included. |
| **Cochrane**  **Search Date:**  2019-03-11 | ((ti.ab.kw.("Hb A1c" OR “A1C” OR “GHb” OR “HbA1c” OR “HbA1” OR “Hb A1” OR ”hemoglobin A1c” OR ”hemoglobin A1c” OR ”haemoglobin A1c” OR ”haemoglobin A1c” OR “glycohemoglobin” OR “glyco hemoglobin” OR “glycohemoglobins” OR “glyco hemoglobins” OR “glycohaemoglobin” OR “glyco haemoglobin” OR “glycohaemoglobins” OR “glyco haemoglobins” OR “hemoglobin A(1)” OR “haemoglobin A(1)” OR “glycosylated hemoglobin” OR “glycosylated hemoglobins” OR “glycosylated haemoglobin” OR “glycosylated haemoglobins” OR “glycated hemoglobins” OR “glycated hemoglobin” OR “glycated haemoglobins” OR “glycated haemoglobin”) OR ("Glycated Hemoglobin A" MeSH descriptor/exp)) AND (ti.ab.kw. (“T2D” OR diabet* OR “T2DM” OR “NIDDM” OR “MODY” OR “IDDM” OR prediabet* OR "pre diabetic" OR "pre diabetes") OR ("Diabetes Mellitus" MeSH descriptor/exp)) AND (ti.ab.kw. (retinopathy OR "retinal disease" OR "retinal diseases" OR “retinal vascular disease” OR “retinal vascular diseases” OR retinopathies OR neuropathy OR neuropathies OR nephropathies OR nephropathy) OR (“Diabetic Retinopathy" MeSH descriptor OR "Retinal Diseases" MeSH descriptor OR “Diabetic Neuropathies" MeSH descriptor OR “Diabetic Nephropathies" MeSH descriptor) AND (ti.ab.kw.(diagnos* OR threshold* OR cutoff OR "cut point" OR cutpoint*OR "cut points" OR "cut off") OR ("Diagnosis" MeSH descriptor/exp)) | **604**  (1 review 603 trials) | All terms searched in the field for title, abstract and keyword (here marked with “ti.ab.kw.”) and in MeSH (here marked with "MeSH descriptor") when available  Publication year filter for 1990- applied  **MeSH/thesaurus variations compared to PubMed:**  None. |
| **Scopus**  **Search Date:**  2019-03-11 | ((TITLE-ABS-KEY ("Hb A1c" OR “A1C” OR “GHb” OR “HbA1c” OR “HbA1” OR “Hb A1” OR ”hemoglobin A1c” OR ”hemoglobin A1c” OR ”haemoglobin A1c” OR ”haemoglobin A1c” OR “glycohemoglobin” OR “glyco hemoglobin” OR “glycohemoglobins” OR “glyco hemoglobins” OR “glycohaemoglobin” OR “glyco haemoglobin” OR “glycohaemoglobins” OR “glyco haemoglobins” OR “hemoglobin A(1)” OR “haemoglobin A(1)” OR “glycosylated hemoglobin” OR “glycosylated hemoglobins” OR “glycosylated haemoglobin” OR “glycosylated haemoglobins” OR “glycated hemoglobins” OR “glycated hemoglobin” OR “glycated haemoglobins” OR “glycated haemoglobin”) AND (TITLE-ABS-KEY (“T2D” OR diabet* OR “T2DM” OR “NIDDM” OR “MODY” OR “IDDM” OR prediabet* OR "pre diabetic" OR "pre diabetes") AND (TITLE-ABS-KEY (retinopathy OR "retinal disease" OR "retinal diseases" OR “retinal vascular disease” OR “retinal vascular diseases” OR retinopathies OR neuropathy OR neuropathies OR nephropathies OR nephropathy) AND (TITLE-ABS-KEY (diagnos* OR threshold* OR cutoff OR "cut point" OR cutpoint*OR "cut points" OR "cut off")) | **71** | All selected search terms searched in the fields for title, abstract and keywords, here marked with “TI-ABS-KEY.”  No thesaurus available.  Publication year filter for 1990- applied |
| **Total no. of references retrieved** | | **12796** | |
| **Total no. of references after de-duplication** | | **9324** | |

**Grey literature**

| **Source and date** | **Search string** | **Result** | **Notes** |
| --- | --- | --- | --- |
| **Source:**  Open Grey  **Search date:**  2019-03-13 | (("Hb A1c" OR “A1C” OR “GHb” OR “HbA1c” OR “HbA1” OR “Hb A1” OR ”hemoglobin A1c” OR ”hemoglobin A1c” OR ”haemoglobin A1c” OR ”haemoglobin A1c” OR “glycohemoglobin” OR “glyco hemoglobin” OR “glycohemoglobins” OR “glyco hemoglobins” OR “glycohaemoglobin” OR “glyco haemoglobin” OR “glycohaemoglobins” OR “glyco haemoglobins” OR “hemoglobin A(1)” OR “haemoglobin A(1)” OR “glycosylated hemoglobin” OR “glycosylated hemoglobins” OR “glycosylated haemoglobin” OR “glycosylated haemoglobins” OR “glycated hemoglobins” OR “glycated hemoglobin” OR “glycated haemoglobins” OR “glycated haemoglobin”) AND (“T2D” OR diabet* OR “T2DM” OR “NIDDM” OR “MODY” OR “IDDM” OR prediabet* OR "pre diabetic" OR "pre diabetes") AND (retinopathy OR "retinal disease" OR "retinal diseases" OR “retinal vascular disease” OR “retinal vascular diseases” OR retinopathies OR neuropathy OR neuropathies OR nephropathies OR nephropathy) AND (diagnos* OR threshold* OR cutoff OR "cut point" OR cutpoint*OR "cut points" OR "cut off")) | **1** | All terms searched in “All fields”  No filters or limitations applied as the databases dates back to 1997- only. |
| **ProQuest Dissertation and Theses**  **Search date:**  2019-03-13 | (("Hb A1c" OR “A1C” OR “GHb” OR “HbA1c” OR “HbA1” OR “Hb A1” OR ”hemoglobin A1c” OR ”hemoglobin A1c” OR ”haemoglobin A1c” OR ”haemoglobin A1c” OR “glycohemoglobin” OR “glyco hemoglobin” OR “glycohemoglobins” OR “glyco hemoglobins” OR “glycohaemoglobin” OR “glyco haemoglobin” OR “glycohaemoglobins” OR “glyco haemoglobins” OR “hemoglobin A(1)” OR “haemoglobin A(1)” OR “glycosylated hemoglobin” OR “glycosylated hemoglobins” OR “glycosylated haemoglobin” OR “glycosylated haemoglobins” OR “glycated hemoglobins” OR “glycated hemoglobin” OR “glycated haemoglobins” OR “glycated haemoglobin”) AND (“T2D” OR diabet* OR “T2DM” OR “NIDDM” OR “MODY” OR “IDDM” OR prediabet* OR "pre diabetic" OR "pre diabetes") AND (retinopathy OR "retinal disease" OR "retinal diseases" OR “retinal vascular disease” OR “retinal vascular diseases” OR retinopathies OR neuropathy OR neuropathies OR nephropathies OR nephropathy) AND (diagnos* OR threshold* OR cutoff OR "cut point" OR cutpoint*OR "cut points" OR "cut off")) | **35** | Search in the field: “Anywhere except full text”  Publication year filter for 1990- applied |
| **The New York Academy of Medicine - Grey Literature Report**  **Search date:**  2019-03-13 | Several different search combination tried with 0 or totally irrelevant references as result | **0** |  |
| **Clinical Trials.gov**  **Search date:**  2019-03-13 | “Diabetes Mellitus” AND "Hb A1c" AND Diagnosis AND (nephropathy OR retinopathy) | **13** | Search filter for “Completed studies” applied. |
| **Total no. of references retrieved** | | **49** | |
| **Total no. of references after de- duplication** | | **46** | |
