# Supplementary material for: Diagnosing type 2 diabetes using Hemoglobin A1c: a systematic review and meta-analysis of the diagnostic cutpoint based on microvascular complications
Source: Acta Diabetol. 2020 Nov 3;58(3):279–300. doi: 10.1007/s00592-020-01606-5 (PMC7907031; doi:10.1007/s00592-020-01606-5)
Supplement: Supplementary file 4 — Summary of precision and risk of bias (ROB) assessment. Summary of precision and risk of bias (ROB) assessment for studies reporting the prevalence of microvascular complications associated with type 2 diabetes mellitus (DOCX 17 kb) [file 592_2020_1606_MOESM4_ESM.docx]

**Supplementary Table 3**. Summary of precision and risk of bias (ROB) assessment for studies reporting the prevalence of microvascular complications associated with Type 2 Diabetes Mellitus.

| **Quality assessment** | **Retinopathy prevalence** | | **Nephropathy prevalence** | | **Neuropathy prevalence** | |
| --- | --- | --- | --- | --- | --- | --- |
|  | **n** | **%** | **n** | **%** | **n** | **%** |
| **Precision of estimates** |  |  |  |  |  |  |
| High precision^a^ | 16 | 72.7 | 2 | 50.0 | 2 | 66.7 |
| Low precision | 6 | 27.3 | 2 | 50.0 | 1 | 33.3 |
| **Risk of bias quality domains** |  |  |  |  |  |  |
| **Consistency in HbA1c diagnostic** ^b^ |  |  |  |  |  |  |
| Low risk of bias | 18 | 81.8 | 4 | 100.0 | 2 | 66.7 |
| High risk of bias | 4 | 18.2 | -- | -- | 1 | 33.3 |
| Unclear^c^ | -- | -- | -- | -- | -- | -- |
| **Sampling methodology**^d^ |  |  |  |  |  |  |
| Low risk of bias | 21 | 95.5 | 4 | 100.0 | 3 | 100.0 |
| High risk of bias | 1 | 4.5 | -- | -- | -- | -- |
| Unclear^*^ | -- | -- | -- | -- | -- | -- |
| **Total number of included studies** | **22** | **100.0** | **4** | **100.0** | **3** | **100.0** |
| **Summary of ROB assessment** |  |  |  |  |  |  |
|  | **n** | **%** | **n** | **%** | **n** | **%** |
| **Low risk of bias** |  |  |  |  |  |  |
| In one quality domain | 21 | 95.5 | 4 | 100.0 | 3 | 100.0 |
| In both quality domains | 18 | 81.8 | 4 | 100.0 | 2 | 66.7 |
| **High risk of bias** |  |  |  |  |  |  |
| In one quality domain | 4 | 18.2 | 4 | 100.0 | 1 | 33.3 |
| In both quality domains | 1 | 4.5 | 0 | 0.0 | 0. | 0.0 |
| **Total number of included studies** | **22** | **100.0** | 4 | 100.0 | 3 | 100.0 |

^a^Studies including 100 or more participants were classified as being of higher precision.

^b^Studies using the same diagnostic tool for measuring HbA1c across all study participants were classified as having low risk of bias.

^c^Studies with missing information for any of the domains were classified as having unclear ROB for that specific domain.

^d^Studies using probability-based sampling for participants’ recruitment as having low risk of bias.
