# Supplementary material for: Diagnosing type 2 diabetes using Hemoglobin A1c: a systematic review and meta-analysis of the diagnostic cutpoint based on microvascular complications
Source: Acta Diabetol. 2020 Nov 3;58(3):279–300. doi: 10.1007/s00592-020-01606-5 (PMC7907031; doi:10.1007/s00592-020-01606-5)
Supplement: Supplementary file 5 — Study-specific quality assessments (DOCX 19 kb) [file 592_2020_1606_MOESM5_ESM.docx]

**Supplementary Table 4.** Study-specific quality assessments.

|  | **Retinopathy** |  | **Precision** | **Risk of bias** | **Risk of bias** |
| --- | --- | --- | --- | --- | --- |
|  | **Authors** | **Title** | **Was the sample size at least 100 for each HbA1c category** | **Was the HbA1c Diagnostic measurement performed using the same methodology for all participants in the study?** | **How was study population recruited?** |
| 1 | Colagiuri et al 2011 | Glycemic thresholds for Diabetic retinopathy | Higher | High ROB | High ROB |
| 2 | Engelgau et al (1997), Egypt | Comparison of fasting and 2-hour glucose and HbA1c levels for diagnosing diabetes | Lower | Low ROB | Low ROB |
| 3 | Ito et al (2000) Japan | Importance of OGTT for diagnosing diabetes mellitus based on prevalence and incidence of retinopathy | Higher | Low ROB | Low ROB |
| 4 | Tapp et al (2006) Australia | Diagnostic thresholds for diabetes: the association of retinopathy and albuminuria with glycemia; AUSDiab Study | Higher | Low ROB | Low ROB |
| 5 | Aidenloo NS (2016) | Optimal glycemic and hemoglobin A1c thresholds for diagnosing diabetes based on prevalence of retinopathy in an Iranian population | Higher | Low ROB | Low ROB |
| 6 | Almdal 2014 | Glycemic threshold for diabetes specific retinopathy among individuals from Saudi Arabia, Algeria and Portugal | Higher | Low ROB | Low ROB |
| 7 | Bertelsen 2014 | Sex differences in risk factors for retinopathy in non-diabetic men and women: the Tromso Eye study | Higher | Low ROB | Low ROB |
| 8 | Bower JK 2013 | No ethnic difference in the Association of glycated hemoglobin with retinopathy | Higher | Low ROB | Low ROB |
| 9 | Cheng YJ 2009 | Association of A1C and fasting plasma glucose levels with diabetic retinopathy prevalence in the US population | Higher | Low ROB | Low ROB |
| 10 | Cho NH 2013 | Optimal HbA1c cutoff for detecting diabetic retinopathy | Higher | Low ROB | Low ROB |
| 11 | Fukushima S, 2013 | Prevalence of retinopathy and its risk factors in a Japanese population | Lower | Low ROB | Low ROB |
| 12 | Selvin 2011 | Glycated hemoglobin and the risk of kidney disease and retinopathy in adults with and without diabetes | Higher | Low ROB | Low ROB |
| 13 | Park YM 2014 | Glycaemic and haemoglobin A1c thresholds for detecting diabetic retinopathy: the fifth Korea national Health and Nutrition Examination (2011) | Lower | Low ROB | Low ROB |
| 14 | Lamparter 2014 | Prevalence and associations of diabetic retinopathy in a large cohort of prediabetic subjects: the Gutenberg Health study | Higher | Low ROB | Low ROB |
| 15 | Metcalf PA 2017 | HbA1c in relation to incident diabetes and diabetes-related complications in non-diabetic adults at baseline | Higher | Low ROB | Low ROB |
| 16 | Okosun IS 2016 | Diagnostic performance of glycated hemoglobin for diabetic retinopathy in non-diabetic older overweight/obese African Americans | Lower | High ROB | Low ROB |
| 17 | Pang 2011 | Determination of diabetic retinopathy prevalence and associated risk factors in Chinese diabetic and pre-diabetic subjects: Shanghai diabetic complications study | Higher | Low ROB | Low ROB |
| 18 | Sabanayagam C 2014 | Diagnosis of Diabetes Mellitus Using HbA1c in Asians: Relationship between HbAc and retinopathy in a multiethnic population | Higher | Low ROB | Low ROB |
| 19 | Sabanayagam C 2009 | Relationship between glycated hemoglobin and microvascular complications: is there a natural cut-point for the diagnosis of diabetes? | Higher | High ROB | Low ROB |
| 20 | Tsugawa Y 2012 | New Diabetes Diagnostic threshold of hemoglobin A1c and the 3-year incidence of retinopathy | Higher | Low ROB | Low ROB |
| 21 | Massin P 2011 | Hemoglobin A1c and fasting plasma glucose levels as predictors of retinopathy at 10 years: the French DESIR study | Lower | Low ROB | Low ROB |
| 22 | Tsugawa Y 2012 | Should the Hemoglobin A1c Diagnostic cutoff differ between blacks and whites? | Lower | High ROB | Low ROB |
|  |  |  |  |  |  |
|  | **Nephropathy** |  |  |  |  |
|  | **Authors** | **Title** | **Precision** | **Risk of bias** | **Risk of bias** |
| 1 | Tapp et al (2006) Australia | Diagnostic thresholds for diabetes: the association of retinopathy and albuminuria with glycemia; AUSDiab Study | Lower | Low ROB | Low ROB |
| 2 | Metcalf PA 2017 | HbA1c in relation to incident diabetes and diabetes-related complications in non-diabetic adults at baseline | Higher | Low ROB | Low ROB |
| 3 | Toulis et al 2018 | Glycated hemoglobin, albuminuria and surrogate markers of macrovascular disease; the Guangzhou Biobank Cohort Study | Lower | Low ROB | Low ROB |
| 4 | Xing et al 2014 | Association of pre-diabetes by fasting glucose and/or HbA1c levels with subclinical atherosclerosis an impaired renal function; observations from the Dallas Heart Study | Higher | Low ROB | Low ROB |
|  |  |  |  |  |  |
|  | **Neuropathy** |  |  |  |  |
|  | **Authors** | **Title** | **Precision** | **Risk of bias** | **Risk of bias** |
| 1 | Metcalf PA 2017 | HbA1c in relation to incident diabetes and diabetes-related complications in non-diabetic adults at baseline | Higher | Low ROB | Low ROB |
| 2 | Kurisu et al (2016) poster | Polyneuropathy or neuropathic pain did not increase at prediabetic stage in a Japanese population | Lower | High ROB | Low ROB |
| 3 | Tapp 2003 | Foot complications in type 2 diabetes: an Australian population-based study. | Higher | Low ROB | Low ROB |
